# Cell cycle in plant development and reprogramming

**DOI:** 10.1242/dev.205318

**Published:** 2026-04-02

**Authors:** Laura R. Lee, Keiko U. Torii

**Affiliations:** ^1^ Department of Biology, University of North Carolina at Chapel Hill, Chapel Hill, NC 27599, USA; ^2^ Howard Hughes Medical Institute and Department of Molecular Biosciences, The University of Texas at Austin, Austin, TX 78712, USA

**Keywords:** Cell cycle, Stomata, Roots, Stem cells, Reprogramming

## Abstract

The development of multicellular organisms relies on controlled cell divisions and differentiation that generate specific cell types of functional tissues and organs. Control of the cell cycle and its checkpoints are tightly intertwined with the maintenance of stem cells, cell fate acquisition and cellular reprogramming. This Review focuses on cell cycle-mediated control of plant development and regeneration, where cell division and differentiation occur in the absence of cell migration. We examine two systems – the root apical meristem and leaf epidermis (stomata) – and explore how master-regulatory transcription factors directly impact the cell cycle to achieve differentiation of specific cell types, as well as how epigenetic machineries guide or constrain such processes. We further emphasize the importance of G_1_ cell cycle phase duration and G_2_/M checkpoints for stem cell differentiation and regeneration. By synthesizing recent discoveries, we aim to highlight cell cycle regulation that underpins both robustness and plasticity of plant development and regeneration. Such knowledge will ultimately enhance our understanding of the commonalities and uniqueness of cell cycle regulation between plants and metazoans.

## Teaser

**Summary:** Focusing on the root and the stomatal lineage, this Review highlights how cell cycle control underpins stem cell maintenance, differentiation and regenerative plasticity in plant development.

## Introduction

During plant development, it is essential that a tight coordination between the cell cycle and developmental processes is maintained. Unlike animal cells, plant cells have stiff cell walls that prevent migration relative to one another, so cell fate and position in plants are determined simultaneously through cell division. As such, the plant cell cycle serves as both a major regulator of development as well as a key integrator of growth and environmental response. Despite this fundamental relationship, studying the plant cell cycle in a developmentally relevant context remains technically challenging. However, techniques developed in recent years, building on earlier foundational work, now enable unprecedented advancement in our understanding of the relationship between cell cycle and plant development. Here, we summarize the current literature on how cell cycle and developmental processes are coordinated, while highlighting new tools and findings that will catalyse further advancements in this field. Finally, we identify key unanswered questions and propose forward-looking priorities for the field.

Plant cell cycle researchers have made significant progress in elucidating core molecular cell cycle regulators in plants (Gutierrez, 2016), defining the functions of multiple classes of cyclins, cyclin-dependent kinases and transcriptional modulators using a combination of biochemistry (John et al., 1989), molecular biology (Dahl et al., 1995; Soni et al., 1995), mutant analysis (Johnston et al., 2010) and synchronized plant cell cultures (Menges and Murray, 2002). Interestingly, whereas many core cell cycle regulators are conserved between plants and animals, some cell cycle regulators are missing or, alternatively, specifically adopted in plants (Gutierrez, 2022; Inzé and De Veylder, 2006). This inspires an interesting research direction that aims to understand how cell cycle machinery is tied into cell proliferation and cell fate specification in plants. Certain division regulation mechanisms are also unique to plants. For example, cytokinesis is completed in plants with the formation of a new cell wall between the two daughters guided by a cytoskeletal structure called the preprophase band (Livanos and Müller, 2019). In animals a contractile actomyosin ring cleaves the mother cell into two daughters (Pollard and O'Shaughnessy, 2019). More recently, technical advances allowed new insights into plant cell cycle regulation across both time and space, leading to the characterization of cell cycle regulation specific to developmental stage (Timilsina et al., 2019) and cell type (Adrian et al., 2015; Han et al., 2018; Kono et al., 2007; Sozzani et al., 2010; Weimer et al., 2018). In particular, the recent advent of molecular biology and genomic approaches have revealed a key feature of cell cycle-development coordination: that cell cycle regulators are often themselves controlled by cell fate-specifying transcription factors (Han et al., 2018; Sozzani et al., 2010; Weimer et al., 2018). These findings, which were enabled in part by transcriptomics and microscopy, underline the tight coordination between cell cycle and cell fate in plant development.

Two technologies of particular note for studying the relationship between the cell cycle and development are cell cycle reporters, which have enabled long-term time-lapse imaging of cell division (Winter et al., 2024), and single cell RNA-sequencing (scRNA-Seq) (Lopez-Anido et al., 2021; Shahan et al., 2022). Multi-colour cell cycle reporters – such as Plant FUCCI (PlaCCI) (Desvoyes et al., 2020) – have made it possible to measure the lengths of individual cell cycle phases using live imaging, enabling novel insights into cell cycle regulation during development (Han et al., 2022) and regeneration (Lee et al., 2025). Leveraging scRNA-seq information has also led to the recent identification of context-specific cell cycle regulation (Lee et al., 2025), as well as asymmetric hormone signalling regulating mitosis (Vukašinović et al., 2025). In this Review, we describe in detail the context and significance of these tools and findings for the study of cell cycle regulation and cell fate specification in root and shoot epidermal development, where stomatal cell lineages are generated.

## Core molecular regulators of cell cycle phases during development

**Fig. 1.**
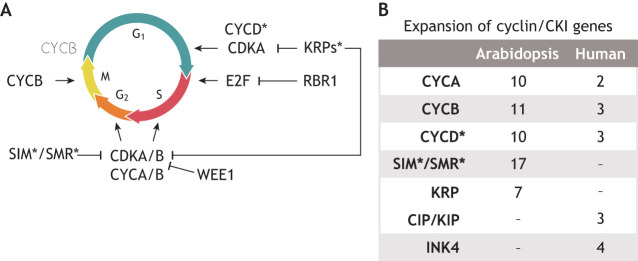
**Conservation and divergence of core cell cycle regulators in plants.** (A) A schematic of the mitotic cell cycle overlaid with core cell cycle regulators at the part of the cell cycle they regulate. Plant-specific regulators are annotated with an asterisk. Cyclin/cyclin-dependent kinase complexes (CYC/CDK) phosphorylate target proteins to drive cell cycle progression. RBR1 (RETINOBLASTOMA RELATED 1) binds to and negatively regulates E2F until RBR1 is phosphorylated by cyclin/CDK, which releases E2F to drive the expression of S-phase genes in the nucleus. The cell cycle is inhibited or slowed by plant-specific KRP (KIP-RELATED PROTEIN) and SIM (SIAMESE)/SMR (SIAMESE-RELATED) protein, which bind to and block the activity of cyclin/CDK complexes, as do other kinases such as WEE1. Degradation of cyclins via the anaphase-promoting complex (APC) is necessary for the completion mitosis, indicated by the dashed CYCB in the top left of the panel (reviewed by De Veylder et al., 2007; Gutierrez, 2022; Inzé and De Veylder, 2006; Simonini, 2025). (B) A summary table of core cyclin and CKI genes in the *Arabidopsis* and human genomes, highlighting the expansion of these gene families in the *Arabidopsis* genome. Cyclin D proteins are marked with an asterisk because they bear limited sequence similarity to mammalian G1 cyclins but strong functional conservation. Similarly, SIM and SMR are marked because they have conserved CKI activity but lack sequence similarity with the human CKIs. Cyclin D proteins control the G1 to S transition in both plants and animals, but show little sequence similarity between the species (De Veylder, 2019; Kumar and Larkin, 2017; Vandepoele et al., 2002; Wang et al., 2004).

Numerous core cell cycle regulators are conserved in plants and metazoans (e.g. humans) (Fig. 1A). Yet, their numbers, specific sub-families and regulation uniquely reflect the lifestyle and cytokinesis of plants. While there is variation in cell cycle regulation across developmental (and environmental and/or stress) contexts in plants, a set of core regulators generally control unidirectional cell cycle progression (De Veylder et al., 2007; Dissmeyer et al., 2010; Inzé and De Veylder, 2006) (Fig. 1A). These include cyclins (CYCs) and cyclin-dependent kinases (CDKs), which act together to license cell cycle phase transitions. In plants, both CYCs and CDKs are large families of genes with multiple subtypes that form different CYC-CDK complexes, some of which specifically regulate cell cycle phase transitions and others that only do so in specific cell types. CDKs become active when bound to CYCs and are then able to phosphorylate their targets.

Distinct CYC-CDK complexes regulate different cell cycle phases and transitions (Fig. 1A). The plant-specific family of CYCDs works in complex with CDKAs to license the G_1_/S transition (Oakenfull et al., 2002; Soni et al., 1995); a CDKA-CYCA complex also contributes to this transition (Nowack et al., 2012; Takahashi et al., 2010). During G_1_ phase, the *Arabidopsis* pRB homologue, RETINOBLASTOMA RELATED 1 (RBR1), physically interacts with the E2F family of transcription factors, inhibiting their activity. Unlike metazoans, plants do not possess canonical cyclin E proteins that regulate the G_1_/S transition (Inzé and De Veylder, 2006) (Fig. 1B). At the G_1_/S transition, RBR1 is inactivated via hyperphosphorylation by CYC-CDK (Boniotti and Gutierrez, 2001). Upon RBR1 inactivation, the E2F transcription factors are released to activate expression of their target genes, which drive S phase progression (Rossi and Varotto, 2002). Interestingly, loss-of-function mutations in all three E2F family genes in *Arabidopsis* confer an overproliferation phenotype similar to the phenotype resulting from *RBR1* knockdown, suggesting that all three E2Fs are required to work in a complex with RBR1 to repress cell cycle progression (Gombos et al., 2023). Remarkably, *e2f* triple mutations impact multiple developmental contexts, such as prolonged proliferation in the stomatal lineage as well as ectopic divisions in normally quiescent cells in the root apical meristem (Gombos et al., 2023), suggesting this function of the E2Fs is employed broadly in the plant. A CYCA-CDKA complex forms during S phase and may specifically regulate the onset of endoreduplication, where DNA replication during S phase occurs without mitosis (Imai et al., 2006). A CDKB-CYCB complex forms during G_2_ phase and then licenses the G_2_/M transition (Boudolf et al., 2004; Vanneste et al., 2011; Xie et al., 2010), with a minor contribution from a CDKA-containing complex (Nowack et al., 2012). At the G_2_/M transition, CDKBs work in complex with CYCs, phosphorylating various target proteins that lead to mitosis. During mitosis, A- and B-type CYCs are then targeted for degradation via ubiquitylation by the anaphase-promoting complex (APC), which is necessary for completion of mitosis (reviewed by Willems and De Veylder, 2022). Highly conserved metazoan CDC25 plays a key role in cell cycle progression, especially at the G_2_/M transition and mitotic entry (Boutros et al., 2006). As a dual phosphatase, CDC25 dephosphorylates CDK and activates the CYC-CDK complexes for cell cycle progression. Notably, a canonical CDC25 counterpart is absent in plants (Boudolf et al., 2006), signifying the uniqueness of the plant core cell cycle machinery (Boudolf et al., 2006). Excitingly, very recent studies unravelled the possibility that BRI1 SUPPRESSOR (BSU)/BSU-LIKE (BSL) phosphatases function as a plant counterpart of CDC25 (Rico-Resendiz et al., 2026 preprint; Tulin et al., 2025).

The activity of these proteins is kept in balance by a cohort of negative regulators, including WEE1-like kinase (WEE1) (Sun et al., 1999), and the plant-specific families of ICK/KRP (INHIBITOR OF CDK/KIP-RELATED PROTEIN) (De Veylder et al., 2001; Lui et al., 2000; Wang et al., 1997) and SMR (SIAMESE-RELATED) (Churchman et al., 2006; Peres et al., 2007) CDK inhibitors. These proteins ultimately reduce the activity of CYC-CDK complexes, which prevents G_1_/S and G_2_/M checkpoint licensing, and slows down the cell cycle. Interestingly, the *Arabidopsis* genome encodes several KRP and SMR genes – 7 and 17, respectively (Kumar and Larkin, 2017) (Fig. 1B). The high copy number of KRP and SMR genes creates an opportunity for acquisition of cell type-specific functions. This is the case for SMR4, which is discussed in a later section of this Review. Many of these are expressed in a cell-type- or developmental stage-specific manner, likely contributing to variation in cell cycle length among developmental contexts. This also mirrors the general trend of a higher copy number of core cell cycle regulators in the *Arabidopsis* genome compared to the human genome (Fig. 1B), which is likely linked to the acquisition of cell type-specific function of cell cycle regulators in plants.

Variation in cell cycle phase length has also been observed to impact cell fate specification (Dalton, 2015). Changes to G_1_ phase duration are highly associated with cell identity changes. In animals, embryonic stem cells (ESCs) have a short G_1_ phase that lengthens as they differentiate and it has been postulated that their fate commitment programs are initiated in G_1_ phase (reviewed by Dalton, 2015; Padgett and Santos, 2020). Variation in G_1_ length has been linked to specific cell fate outcomes (Jang et al., 2019) and G_1_ length has been mechanistically tied to the accumulation of cell fate-enforcing histone modification H3K27me3 (Trouth et al., 2025). However, other work indicates that overall cell cycle length, rather than G_1_ phase specifically, controls cell fate in animals (reviewed by Padgett and Santos, 2020) and it is possible that this relationship may be context specific. In plants, the relationship between stemness and cell cycle phase duration varies between contexts and, in some cases, is explicitly due to the activity of KRP or SMR proteins. For example, cell size variability within the *Arabidopsis* shoot apical meristem can be normalized by the length of G_1_ phase preceding DNA synthesis – through the amount of KRP4 proteins bound to mitotic chromosomes, both of which are equally inherited from the mother cell (D'Ario et al., 2021). In the *Arabidopsis* root, a G_1_ phase duration gradient exists, in which cells closer to terminal differentiation progress through G_1_ phase faster. This change in G_1_ length has been linked to the activity of KRP5 (Echevarría et al., 2025). In comparison, terminal differentiation in the stomatal lineage is linked to a longer G_1_ phase due to the activity of SMR4 (Han et al., 2022) (see later sections).

Other cell cycle phases are also implicated in fate commitment. In the *Arabidopsis* sepal, giant cells are specified in G_2_ phase when a threshold level of the giant cell-specifying transcription factor (TF), ATML1, is reached. While this is not strictly dependent on G_2_ length, LGO (LEGO)/SMR1 acts downstream of ATML1 during G_2_ phase to trigger endoreduplication and initiate giant cell fate acquisition (Meyer et al., 2017). S phase duration has also been implicated in cell fate specification in animals (Arai et al., 2011), although some evidence suggests replication fork speed is more crucial for cell fate specification than S phase duration itself (Nakatani et al., 2022). Interestingly, the PRC2 complex interacts with DNA replication fork machinery in *Arabidopsis* with consequences for repressive histone modification maintenance (Jiang and Berger, 2017; Yang et al., 2017). Repressive histone modifications catalysed by PRC2 are important for cell fate commitment in *Arabidopsis*, including both roots and stomatal cell lineages (Ikeuchi et al., 2015; Kim et al., 2022; Lee et al., 2019; Lodha et al., 2013; Mozgova et al., 2015). Therefore, it is also possible that replication fork dynamics also influence cell fate maintenance in plants.

## Role of cell cycle in root and stomatal development

In this section, we survey recent advances in the literature about how cell cycle regulation also regulates developmental processes in the root and shoot. Both developmental contexts have unique properties that allow for in depth characterization of the relationship between developmental transitions and cell cycle regulation.

### Root development

**Fig. 2.**
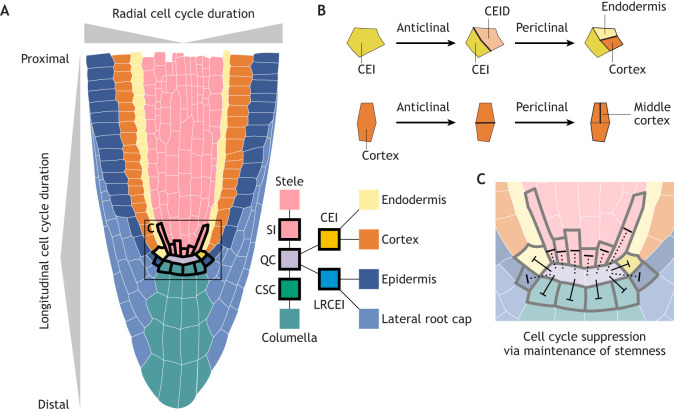
**Coordinated differentiation and cell cycle length gradients in the *Arabidopsis* root.** (A) A schematic of the *Arabidopsis* root is shown, with cell types specified by distinct colours, as defined in the key (bottom right) arranged in clonal vertical files. Cell cycle durations on the radial (top) and proximo-distal (left) axes are indicated in grey. The width of the bar corresponds to the overall length of the cell cycle. The cell cycle in the *Arabidopsis* root is regulated based on both cell lineage and differentiation state. For example, cells in the vasculature divide quickly compared to the cells in the outer files. Conversely, cells close to or in contact with the quiescent centre (QC) divide slowly, while cells that are further displaced from the QC in the proximal direction divide more quickly, which coincides with a more differentiated state. The diagram only covers the root meristematic zone, where the differentiation of mature vasculature and other cell files has not yet occurred (Motte et al., 2019; Rahni and Birnbaum, 2019; Sablowski and Gutierrez, 2022). (B) Division plane orientation is coordinated with cell fate specification in the *Arabidopsis* root. The majority of divisions in the root occur in the anticlinal orientation, which generally results in daughters with the same identity. Here, we highlight two examples of how divisions in, first, the anticlinal orientation and, next, the periclinal orientation can result in new cell identity specification (top) or expansion of files with the same cell identity (bottom). In the example of new cell identity specification (top), the cortical endodermal initial (CEI) first divides anticlinally to repopulate itself and produces the cortical endodermal initial daughter (CEID). That CEID then divides periclinally to produce the endodermis and the cortex. In the second example of file expansion, middle cortex formation is shown. In the distal region of the *Arabidopsis* root apical meristem (RAM), the cortex consists of a single cell layer that propagates through anticlinal divisions. In the proximal, more mature, RAM, the division plane can switch to allow the formation of the middle cortex, thereby increasing the number of cortical cell files (Cui, 2016; Sablowski and Gutierrez, 2022). (C) The *Arabidopsis* root features a closed meristem that undergoes indeterminate growth. The QC inhibits differentiation of columella stem cells (CSCs), which in turn inhibits the cell cycle in the CSCs that are in contact with the QC. This well-supported relationship is shown with solid lines. Some evidence suggests the QC also prevents differentiation in other RAM stem cells. This more hypothetical relationship is illustrated with dotted lines (Strotmann and Stahl, 2021). LRCEI, lateral root cap endodermal initial; SI, stele initial.

As the premiere plant model system, the majority of cell cycle characterization in the root has been performed in *Arabidopsis*. Here, we focus on studies from that species. The root is separated into zones based on developmental status: the meristematic zone (Fig. 2A), where cells actively divide; the elongation zone, where cells begin to elongate and endoreduplicate; and the differentiation zone, where cells adopt mature morphology (reviewed by Petricka et al., 2012). In the meristematic zone, the root apical meristem (RAM) contains a population of stem cells that fuel the indeterminate growth of the root (Dolan et al., 1993; Petricka et al., 2012). The RAM consists of actively dividing cells at the root tip, including stem cells and a central population called the quiescent centre (QC). In the *Arabidopsis* root, a group of 4-6 cells makes up the QC (Dolan et al., 1993) (Fig. 2A). These cells divide less than once per week in 3- to 5-day-old post-germination seedlings (Rahni and Birnbaum, 2019) and evidence suggests these divisions occur more frequently in the RAM of more mature seedlings (Timilsina et al., 2019). Stem cell initials, which are fate-specified stem cells undergoing asymmetric divisions, surround the QC (Fig. 2A). Peripheral to the initials are cells that are further along in the process of differentiation and undergo faster divisions (Echevarría et al., 2025; Rahni and Birnbaum, 2019; Strotmann et al., 2025). Here, we describe how the relationship between cell cycle control and cell identity contributes to the strict patterning of the RAM and the relationship between differentiation and cell cycle speed in this organ.

A striking aspect of the *Arabidopsis* RAM is that cell fate is specified as early as one division away from the central population of stem cells, highlighting the tight spatial regulation of cell identity in the organ (Petricka et al., 2012). Moreover, the *Arabidopsis* RAM is patterned such that there is spatial separation between cells based on cell type, developmental stage and cycling behaviour; this organization of cell identity and proliferation speed is distinct from other *Arabidopsis* organs (reviewed by Uchida and Torii, 2019). As such, the RAM is an organ in which cell division and cell fate specification are uniquely linked relative to the rest of the plant.

The spatial patterning in the RAM provided early insights into the relationship between cell identity, signalling and cell division. It has been proposed that the QC functions as an organizing centre (van den Berg et al., 1997) or as one component of a more distributed signalling system that controls differentiation in the RAM (Rahni et al., 2016). For example, it is well-documented that the QC influences differentiation status of the neighbouring columella stem cells (CSCs) (van den Berg et al., 1997). This may be controlled via cell-cell mobility of a transcription factor expressed in the QC, homeobox gene WOX5 (WUSCHEL-RELATED HOMEOBOX5) (Pi et al., 2015; Strotmann et al., 2025), although it has also been suggested that it is not WOX5 per se but some other QC-derived signalling molecule (Berckmans et al., 2020). In the QC itself, WOX5 maintains quiescence by directly repressing the expression of CYCD genes (Forzani et al., 2014).

One consequence of this inhibition of differentiation is an enforcement of slow cell cycle speed (Fig. 2C). The specific loss of the QC removes this differentiation inhibition, which increases cell division activity in the CSCs and in the CEI (Barlow, 1974; van den Berg et al., 1995), although wounding response may also contribute to increased cell division in the case of QC ablation. Consistent with the function of the QC as a negative regulator of cell division by inhibiting differentiation, cell cycle speed increases as cells are displaced proximally from the stem cell initials via division (Otero et al., 2016; Rahni and Birnbaum, 2019). This change in division speed is driven largely by a G_1_ phase duration gradient that is enforced by a mirrored gradient of PLETHORA (PLT) expression (Aida et al., 2004; Echevarría et al., 2025). Blocking symplastic cell-to-cell connections between the QC and the surrounding initials also causes ectopic divisions and alters the PLT gradient (Liu et al., 2017). Non-cell autonomous regulation may also impact differentiation and cell division in the vascular initials (VIs). WOX5 is present in the vascular initials where it exists in a complex with BRAVO (BRASSINOSTEROIDS AT VASCULAR AND ORGANIZING CENTER) (Strotmann et al., 2025). BRAVO suppresses cell divisions (Vilarrasa-Blasi et al., 2014), and BRAVO expression in the proximal meristem decreases in WOX5 mutants (Betegón-Putze et al., 2021). These findings suggest the presence of diverse mechanisms through which RAM organization and cell cycle progression are linked.

Conversely, cell cycle regulation is also crucial for cell fate specification in the RAM. Cells in the RAM are organized by cell type in concentric files or in longitudinal layers of cells with the same identity (Fig. 2A) (Dolan et al., 1993; Petricka et al., 2012). The files are populated by stem cell initials that contact the QC via infrequent divisions that occur anticlinally, meaning perpendicular to the major growth axis (Fig. 2A,B). Under specific circumstances, the division plane switches to a periclinal orientation, which is parallel to the growth axis, resulting in the expansion of the number of cell files and the specification of a new cell identity. A classic example of this is the establishment of the cortex and endodermal cell files. These neighbouring cell files are born from the same stem cell initial: the cortical endodermal initial (CEI) (Dolan et al., 1993) (Fig. 2B). This cell undergoes an anticlinal, asymmetric division to repopulate itself and produce the cortical endodermal initial daughter (CEID). In the CEID, the division plane switches to periclinal and two cell files are formed. This formative division is regulated by cell type-specific expression of D-type cyclin: CYCD6;1 (Sozzani et al., 2010). The CYCD6;1 locus is under direct control of SHR (SHORT ROOT) and SCR (SCARECROW) (Sozzani et al., 2010), which are transcription factors required for specifying cortex and endodermal fate (Di Laurenzio et al., 1996; Helariutta et al., 2000).

Further linking cell cycle and fate in this context, RBR1 represses SHR-SCR activity to control the spatial patterning of asymmetric cell divisions in the root (Cruz-Ramírez et al., 2012). The knockdown of RBR1 triggers asymmetric cell division of the QC, resulting in excessive columella cell layers (Cruz-Ramírez et al., 2013). Dysregulation of the cell cycle in the RAM, in turn, impacts root development, thus highlighting the crucial role of the cell cycle for organ growth (Cruz-Ramírez et al., 2013), which may function via direct cell cycle regulation, as well as the role of RBR1 in cell fate maintenance. As such, the formation of these files exemplifies the coupling of cell fate specification and cell type-specific expression of a core cell cycle regulator. Recent work has revealed that SHR can drive proliferative or formative divisions in the RAM, depending on the cell-cycle phase in which a minimum threshold of SHR protein is reached (Winter et al., 2024), further tying cell fate specification to the cell cycle. Other division plane switches paired with cell fate changes include middle cortex formation (Fig. 2B) (reviewed by Cui, 2016) and the establishment of lateral root primordia (reviewed by Péret et al., 2009).

While zones of high and low division speed in the root have long been recognized, the function of slow divisions in the QC and immediately surrounding initials remains unclear. Several lines of evidence suggest that the QC is protected from DNA damage by its slow divisions (Burian et al., 2016; Clowes, 1959; Fulcher and Sablowski, 2009; Heyman et al., 2013; Vilarrasa-Blasi et al., 2014). Thus, one possibility is that maintaining slow divisions and an overall low number of divisions in the QC is a protective adaptation to safeguard the genome of the plant stem cell reservoir. However, conflicting data suggest that time, rather than division count, is actually the crucial determinant of mutation accumulation (Satake et al., 2023). This potential trade-off is especially relevant to the biological phenomenon of regeneration. The RAM can regenerate from fate-specified cells following complete excision of all stem cells and the QC (Sena et al., 2009). During regeneration, cells divide three times faster than during homeostatic growth (Rahni et al., 2026). These fast divisions occur due to a truncated G_1_ phase that is coordinated by a sudden influx of the metabolite glutathione in the nuclei of cells in G_1_ phase (Lee et al., 2025). As such, the remaining cells in the RAM can toggle between slow and fast cell division states. One possibility is that slow cell divisions are favourable during homeostatic growth to maintain genome integrity in the stem cell initials, but fast divisions occur during regeneration to quickly repair tissue damage. As such, the ability to toggle division speed could represent a bet-hedging mechanism in fate-committed RAM cells (Lee et al., 2025).

## Epidermal and stomatal development

The shoot epidermis is the outermost single cell layer that guides growth and serves as the interface of a plant and the environment (Glover, 2000). The shoot epidermis of photosynthetic organs, such as leaves, are composed of different cell types, each reflecting their unique function. Briefly, pavement cells that ‘pave’ the epidermis accumulate thick cuticles to protect plants from a diverse array of environmental stresses, including drought, flood and ultraviolet radiation (Zuch et al., 2022). Pavement cells are highly interlocked so that the epidermis can withstand tensions generated by the growth and expansion of internal tissues. Dispersed among pavement cells are stomata (singular stoma): adjustable valves composed of a pore surrounded by paired guard cells that facilitate gas exchange for efficient photosynthesis while minimizing water loss (Zuch et al., 2022). Additionally, the shoot epidermis produces trichomes, which are branched or gland-like cellular appendages with diverse structure and function, among other cell types (e.g. bulliform cells in maize leaf) (Robinson and Roeder, 2015; Torii, 2021). Herein, we focus on developmental trajectories leading to the generation of pavement cells and stomata in *Arabidopsis* shoots.

**Fig. 3.**
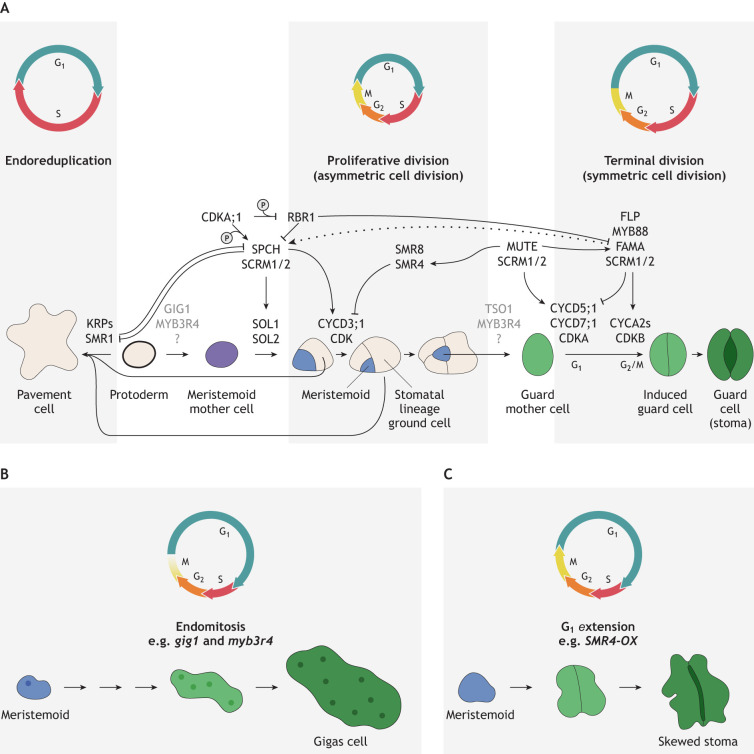
**The role of the cell cycle during leaf epidermal and stomatal development.** (A) Schematic diagram of epidermal and stomatal cell-state transition with specific cell cycle modes depicted (top). A protodermal cell may differentiate directly into a pavement cell, where the cyclin-dependent kinase inhibitors KRP (KIP-RELATED PROTEIN) and SMR1 (SIAMESE-RELATED 1) protein play key roles in initiating endoreduplication. Grey text for GIG1 (GIGAS CELL1) and Myb-domain protein 3R4 (MYB3R4) implies possible regulation. With high activity of SPCH (SPEECHLESS) and SCRM1/2 (SCREAM1/2), the meristemoid mother cell initiates fast asymmetric cell division cycles. The cyclin-dependent kinase CDKA;1 is known to phosphorylate SPCH and increase its stability. The cells with high activities of SPCH-SCRM1/2 will adopt stomatal-lineage fate. Stomatal-lineage ground cells (SLGCs) with high SMR1 activities will differentiate into pavement cells. Upon differentiation to guard mother cells, the MUTE-SCRM1/2 module switches from asymmetric cell division cycles to a slow terminal symmetric division cycle by inducing SMR4 (and SMR8), which inhibits CYCD3;1 but permits CYCD5;1 (and CYCD7;1)-CDKA to initiate the symmetric division cycle. MUTE also directly induces FAMA and FLP (FOUR LIPS), which inhibit cell cycle progression beyond the single round of G_2_/M transition. The dotted line from FAMA to SPCH shows a possible pathway to fate reversion (see Fig. 5). Reciprocally, both SPCH and FAMA are directly regulated by cell cycle components. Additionally, SPCH-induced SOL1 and SOL2 are crucial to coupling cell division and cell fate within the stomatal precursor cells. (Han and Torii, 2019; Lee and Bergmann, 2019). (B) Dysregulation of cell cycle impacts stomatal cell fate. In the absence of G_2_/M checkpoint regulators GIG1 and MYB3R4, stomatal precursor cells undergo endomitosis, resulting in large abnormal cells, named gigas cells, that express guard cell markers. (Iwata et al., 2011). (C) Precocious expression of SMR4 in an early stomatal precursor cell extends the G_1_ phase, causing meristemoid cell expansion, resulting in a stoma with pavement cell-like guard cells. (Han et al., 2022). MYB88, Myb-domain protein 88; RBR1, RETINOBLASTOMA RELATED 1.

Pavement cells and stomata both originate from protodermal cells in the outermost cell layer (‘L1’) of the leaves (Glover, 2000; Zuch et al., 2022). However, their differentiation involves distinct cell cycle modes (Fig. 3A). Pavement cell morphogenesis is associated with endoreduplication (endocycle), in which DNA replication during S phase occurs without mitosis, resulting in higher ploidy (∼16C) (Edgar et al., 2014; Melaragno et al., 1993). In contrast, stomatal lineage cells undergo mitosis throughout their cell-state transitional events from initial precursors to terminally differentiated guard cells. A stereotypical sequence of cell-state transitions, each characterized by a unique cell cycle mode, has made stomatal development a tractable model for studying how lineage-specific transcription factors and cell cycle machineries are tightly coupled to generate functional epidermal tissues with optimal patterning of stomata (Han and Torii, 2019; Lee and Bergmann, 2019) (Fig. 3A). A subset of protodermal cells, known as meristemoid mother cells (MMCs), accumulate the master regulatory transcription factor SPEECHLESS (SPCH) and undergo asymmetric cell divisions that give rise to a meristemoid and its sister cell called a stomatal-lineage ground cell (SLGC) (MacAlister et al., 2007; Pillitteri et al., 2007) (Fig. 3A). SPCH is a potent inducer of cell division and directly activates the expression of CYCD3;1 and CYCD3;2, G1 cyclins that promote leaf epidermal cell proliferation (Dewitte et al., 2007; Lau et al., 2014) (Fig. 3A). Consequently, persistent high SPCH activity in the meristemoid sustains its ability to reiterate asymmetric amplifying divisions (Fig. 3A). SPCH not only regulates but is also regulated by cell cycle components. CDKA;1 phosphorylates and stabilizes the SPCH protein, whereas RBR1 binds to the *SPCH* gene promoter and regulates its expression (Weimer et al., 2012; Yang et al., 2015). Thus, as with CEI cells in the context of root development (see previous section), the antagonistic interplay of CDKA;1 and RBR1 also influences formative divisions within the stomatal cell lineages. Additionally, SPCH may directly promote cell cycle transition via induction of SOL1 and SOL2 (SUPPRESSOR OF LLP1 1 and 2), the mammalian homologues of which regulate cell-cycle specific gene expression (Simmons et al., 2019).

The sister cell of the meristemoid, SLGC, possesses bipotency: it can either adopt pavement cell fate or re-initiate asymmetric spacing divisions to create a satellite meristemoid (Fig. 3A). Reflecting this bipotency, genes promoting formative divisions are highly expressed in SLGCs, most notably *CYCD3;1*, *CYCD3;2* and *CYCD6;1*, as well as components of the G_2_/M checkpoint (e.g. *CDC20* genes and *FZR3/CCS52B*) that are associated with endoreduplication (Ho et al., 2021). The path from SLGC to pavement cell requires SMR1, a cell cycle inhibitor that terminates the self-renewing potency of SLGCs, likely by inhibiting CDKB1-CYCA2 activity (Dubois et al., 2023; Zuch et al., 2022). The degradation level of SPCH protein, which correlates with the size of SLGCs, likely governs the decision of SLGC to terminally differentiate into the pavement cell (Fung et al., 2025).

A dramatic change in cell cycle regulation occurs during the cell state transition from meristemoid to guard mother cell (GMC), a step orchestrated by MUTE (Pillitteri et al., 2007). Unlike the stem cells within the shoot apical meristem, where cell size is normalized by flexible tuning of G_1_ phase lengths (D'Ario et al., 2021), asymmetric amplifying divisions of meristemoids with short G_1_ phase lengths progressively reduce cell size. The smaller meristemoid cell size correlates with the onset of *MUTE* expression, which likely involves elusive nuclear mechanical stress (Gong et al., 2023). Studies using the cell cycle indicator PlaCCI have revealed that the single terminal symmetric division of GMCs is substantially slower than asymmetric amplifying divisions of meristemoids (Han et al., 2022). MUTE directly induces the expression of SMR4, which preferentially associates with and inhibits CDKA-CYCD3 to clear the path for the slow GMC terminal division driven by G1 cyclins CYCD5;1 and CYCD7;1 (Han et al., 2018; Weimer et al., 2018; Zuch et al., 2023) (Fig. 3A). Unlike SMR1, SMR4 does not induce endoreduplication but instead extends the G_1_ phase. That proliferating meristemoids exhibit a shorter G_1_ phase than differentiating GMCs echoes the cell cycle regulation observed in metazoan (e.g. mammalian) development, where stem cells exhibit a shorter G_1_ phase (Coronado et al., 2013; Salomoni and Calegari, 2010).

The terminal GMC symmetric division generates a stoma with paired guard cells (GCs) that can function as a conduit for gas and water. It is therefore essential that GMCs divide only once. The single round of cell division in GMCs is ensured by the incoherent feed-forward loop initiated by MUTE: MUTE directly induces a suite of cell cycle genes, including *CYCD5;1*, *CDKB1;1* and CYCA2 genes*,* as well as master transcription factors FAMA and FOUR LIPS (FLP) that directly repress these cell cycles genes to restrict the cell division (Hachez et al., 2011; Han et al., 2018; Xie et al., 2010). The CDKB1;1-CYCA2 complex drives the G_2_/M transition of the GMC terminal division (Boudolf et al., 2004). Consequently, triple loss-of function mutations for *cyca2;1 cyca2;2 cyca2;3* – as well as dominant-negative CDKB1;1 – produce round, singular guard cells (Boudolf et al., 2009; Vanneste et al., 2011) (Fig. 3A). Conversely, loss-of-function mutations in *FAMA* or *FLP* (together with its paralog *MYB88*) confer GMC tumours with supernumerary symmetric divisions due to failed withdrawal from the cell cycle (Lai et al., 2005; Ohashi-Ito et al., 2006). After the single symmetric division, GCs stay terminally differentiated, likely through cell cycle withdrawal to G_0_ (Zuch et al., 2023) and chromatin-based repression of stomatal lineage regulatory loci (Lee et al., 2019).

Genetic manipulation of cell cycle components can cause failures in epidermal cell identity specification. On rare occasions, a loss-of-function mutant of *GIGAS CELL1* (*GIG1*), which encodes a negative regulator of APC/C, produces abnormally enlarged epidermal cells expressing guard cell markers (Iwata et al., 2011) (Fig. 3B). Additionally, precocious expression of *SMR4* decelerates cell cycles, resulting in asymmetric cell divisions, causing the formation of large, skewed stomata with pavement cell-like appearance (Han et al., 2022) (Fig. 3C). These abnormal cells with mixed identities caused by cell cycle manipulation highlight that cell cycle and cell fate are tightly interwoven to elaborate the differentiation of specific epidermal cell types.

How transcription factors and cell cycle machinery interplay with epigenetic regulators to specify cell fate commitment and differentiation remains an important question. Histone modifications play crucial roles in the commitment stage from meristemoids to GMCs: it has been shown that MUTE (MUTE-SCRM heterodimers), but not SPCH (SPCH-SCRM heterodimers), specifically associates with BPC1/2 (BASIC PENTACYSTEINE1). Subsequently, BPC1/2 recruits the PRC2 complex to MUTE target loci and deposit local repressive histone marks of H3K27me3 (Kim et al., 2022). As such, during stomatal differentiation, the *SPCH* locus can be repressed in a timely manner. A more recent study shows that FAMA-SCRM associates with chromatin remodelling complex SWI/SNF as well as histone acetyltransferase HAC1 (HISTONE ACETYLTRANSFERASE OF THE CBP FAMILY 1) to regulate proper GC differentiation (Liu et al., 2024). Importantly, as a transition from the MUTE-to-FAMA steps occurs within a single round of the cell cycle (Zuch et al., 2023), these studies emphasize that cell cycle phase-specific epigenetic regulation, both histone modifications and chromatin remodelling, underlie how transcription factors specify cell fate.

In addition, DNA methylation impacts stomatal development: loss-of-function mutants lacking 5-methylcytosine DNA glycosylase/lyase *DME* (*DEMETER*), a paralog of *DME*, *ROS1*, as well as higher-order mutants of *ROS1* with *DME-LIKE2* (*DML2*) and *DML3* overly produce stomatal-lineage cells (Kim et al., 2021; Yamamuro et al., 2014). This is likely due to the transposon-induced DNA methylation in the promoter region of *EPF2*, which encodes a secreted peptide hormone inhibiting SPCH proteins (Yamamuro et al., 2014). As such, the effects of DNA methylation of cell cycle may be indirect.

## Role of cell cycle in root and stomatal-lineage reprogramming

One of the most fascinating aspects of plants is their ability to reprogram damaged organs from previously fate-specified cells, whether via direct trans-differentiation or indirect dedifferentiated-redifferentiation steps (e.g. callus). Regeneration requires cells to dynamically regulate and redefine cell fate and cell cycle state. The interplay of master-regulatory transcription factors, chromatin state and cell cycle control likely underpins these cellular reprogramming events. We address the current state of understanding about these intricate processes in the following section.

### Root cell reprogramming

The *Arabidopsis* root apical meristem is spatially arranged by cell type, differentiation status and cell cycle status, it is an exceptionally well-suited system for studying regeneration (Matosevich and Efroni, 2021; Sena and Birnbaum, 2010). It is possible to determine the identity and differentiation status of a cell based on its relative position in the organ; watching the behaviour of a given cell over time makes it possible to observe both cell cycle behaviour and cell identity transitions. Recent advancements in time lapse microscopy and scRNA-seq have improved our ability to decipher the relationship between the cell cycle and cell fate specification in this organ.

**Fig. 4.**
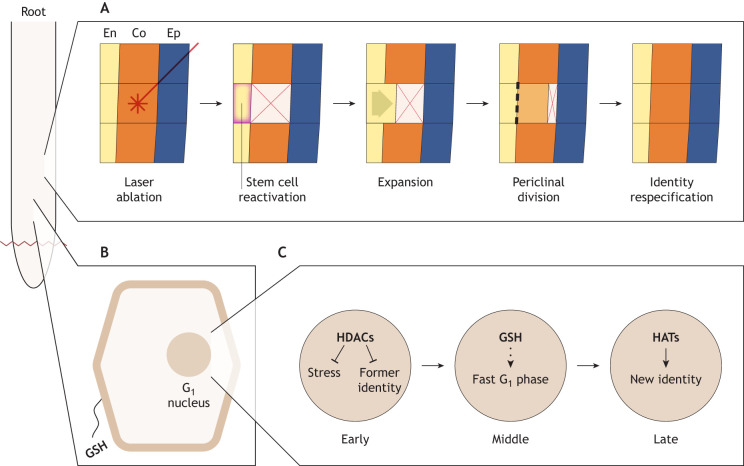
**Regeneration and reprogramming in the *Arabidopsis* root apical meristem.** (A) Targeted laser ablations of individual cells in the root revealed that cells from a more central file expand in the orientation of the lysed cell and then undergo an asymmetric periclinal division to produce one daughter cell in with the fate of the original cell type and a second daughter cell with the fate of the ablated cell type. En, endodermis; Co, cortex; Ep, epidermis (Marhava et al., 2019). (B) In more distally located cells, glutathione (GSH) is released from lysed cells following tissue damage. This then preferentially accumulates in G_1_ phase cell nuclei, which initiates a coordinated cell cycle response to injury (Lee et al., 2025). (C) The stages of events that occur within the nucleus to facilitate identity change during regeneration are shown. In the early phase, histone deacetylases (HDACs) repress gene expression associated with the stress response and the pre-injury cell identity. Cells then go through a period of rapid proliferation triggered by nuclear GSH influx. Finally, histone acetyltransferases (HATs) activate the expression of genes associated with the new cell identity (Lee et al., 2025; Rahni et al., 2026).

An early study found that there is an increase in the number of cell divisions apparent in single snapshot images across multiple cell types during regeneration (Sena et al., 2009). The study further showed that cell division is required for root tip regeneration (Sena et al., 2009). Later, targeted laser ablations in the root tip were used to demonstrate that fate-specified cells accelerate the cell cycle and switch division plane from anticlinal to periclinal to replace the lost cells, after which the daughters of those divisions take on distinct and correctly patterned fates (Fig. 4A) (Marhava et al., 2019). These data support a tight relationship between cell division and cell fate re-specification during regeneration in the root.

Expanding on these observations, a combination of time lapse microscopy and scRNA-seq demonstrated that cell division mediates the acquisition of new cell identities during regeneration (Fig. 4B) (Rahni et al., 2026). Tying this observation to a cell cycle regulatory mechanism, recent work employing long-term light sheet timelapse microscopy and single cell RNA-seq has demonstrated that the rapid cell cycles observed previously during regeneration (Sena et al., 2009) are mediated by G_1_ phase truncation (Lee et al., 2025). This occurred after an influx of glutathione into the nuclei of cells in G_1_ phase, which fits with previous evidence suggesting high nuclear glutathione is required for cells to license the G_1_-to-S phase transition (Diaz Vivancos et al., 2010; Vernoux et al., 2000). Furthermore, cells with the shortest G_1_ phase seem to reprogram the most efficiently (Lee et al., 2025), an observation discovered using long-term time lapse imaging with the multi-colour PlaCCI reporter (Desvoyes et al., 2020). Taken together, these data support a model wherein cells in the root sense damage and respond by activating a unique cell cycle program that allows rapid injury response via tissue repatterning.

## Stomatal-lineage cell reprogramming

**Fig. 5.**
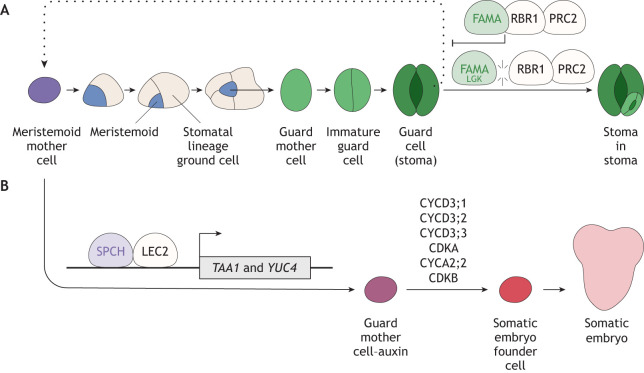
**The role of the cell cycle in reprogramming stomatal-lineage cells.** (A) Reprogramming of guard cells to the stomatal initial cell. Expression of mutant FAMA proteins (FAMA^LGK^) that cannot associate with (and thus failed to be regulated by) RBR1 (RETINOBLASTOMA RELATED 1) results in spontaneous reversion of mature guard cells to reinitiate asymmetric cell division. This results in a striking stoma-in-stoma phenotype. See also Fig. 3A for details of how RBR1 regulates stomatal cell lineages. (Lee et al., 2019; Matos et al., 2014). (B) Reprogramming of stomatal lineage cells to totipotency. The ectopic overexpression of LEC2 (LEAFY COTYLEDON2), in conjunction with SPCH (SPEECHLESS), activates the expression of auxin biosynthesis genes *YUC4* and *TAA1*. The high endogenous auxin accumulation deviates the stomatal-lineage trajectory to a new guard mother cell-auxin state, with elevated expression of CYCD3 (cyclin D3) proteins (G1 cyclins for proliferating epidermal cells) and their partner cyclin-dependent kinase A (CDKA), as well as cyclin A2 (CYCA2) proteins and cyclin-dependent kinase B (CDKB). These cells adopt a somatic embryogenesis founder cell state and eventually regenerate somatic embryos (Tang et al., 2025). PRC2, polycomb repressive complex 2.

While mature stomatal guard cells (GCs) are considered ‘terminally differentiated’, ectopic overexpression of cell cycle components in GCs (including CDKA;1, CYCD5;1, CYCD7;1, CYCD3;2 and CYCA2;3) triggers extra divisions of guard cells (Han et al., 2018; Weimer et al., 2018; Yang et al., 2014). This suggests that mature GCs have the potential to overcome cell cycle arrest and reinitiate mitotic state, despite morphological specialization. What, then, is the mechanism maintaining GCs as terminally differentiated and mitotically inactive? Remarkably, direct association of RBR1 with FAMA appears to be the key (Fig. 5A) (Matos et al., 2014). A mutated FAMA variant that is otherwise functional but can no longer associate with RBR1, FAMA^LGK^, exhibits a striking ‘stoma-in-stoma’ phenotype, where a mature GC re-initiates MMC identity to reiterate stomatal cell-state transitions. RBR1 is known to associate with the components of the PRC2 complex, which then deposit H2K27me3 marks (Gutzat et al., 2011). The inability of FAMA^LGK^ to associate with RBR1 results in a failure to establish repressive histone modifications at shared FAMA and SPCH transcriptional targets (Lee et al., 2019; Matos et al., 2014) (Fig. 4A). Further GC-specific profiling of transcriptomes and histone modifications has revealed that the pro-reprogramming gene *WIND3* is transcriptionally repressed in FAMA^LGK^ guard cells, suggesting differentiated epidermal cells can sense and limit inappropriate reprogramming (Lee et al., 2019).

Among the leaf epidermal cell types, stomatal precursors and mature GCs remain 2C (i.e. somatic nuclear content as a diploid) (Seller and Schroeder, 2023; Torii, 2021). Thus, we could envision that, unlike endoreduplicated pavement cells (∼ 16C) or trichomes (∼ 64C) (Melaragno et al., 1993), stomatal precursor cells might be able to revert back to a totipotent state, which is the highest level of stem cell potency. Indeed, a recent study reports that ectopic overexpression of the transcription factor LEAFY COTYLEDON2 (LEC2) can reprogram a stomatal precursor cell to totipotency, giving rise to a somatic embryo (Fig. 5B) (Tang et al., 2025). It is well established that *LEC2* overexpression confers ectopic somatic embryogenesis on the *Arabidopsis* cotyledon and leaf surfaces (Stone et al., 2001, 2008). Overexpressed LEC2 associates with SPCH; this LEC2-SPCH module directly induces the auxin biosynthesis genes *YUCCA4* (*YUC4*) and *TAA1*, thus increasing auxin levels. Consequently, high accumulation of auxin triggers the deviation of the stomatal differentiation trajectory into a previously unreported ‘GMC-auxin’ state, which eventually adopts the identity of a somatic embryo founder cell (SEFC) (Fig. 5). Reflecting their totipotency, the GMC-auxin cells highly express G1 cyclins that promote cell proliferation: CYCD3 genes and their binding partner CDKA genes, as well as CYCA2 genes and their binding partner *CDKB* (Tang et al., 2025) (Fig. 5). *CYCD3;1* and *CYCD3;2* are direct SPCH targets that normally promote asymmetric cell divisions of MMCs and meristemoids (Adrian et al., 2015). In the GMC-auxin cells, both *SPCH* and *MUTE* are downregulated, whereas the transcription factor gene *BPC1* is upregulated. Normally, BPC1 associates with MUTE to achieve a cell-state transition through repressing the *SPCH* locus through modification of local repressive histone marks (Kim et al., 2022). In the presence of the LEC2-SPCH module, the regulatory circuits of stomatal differentiation become dysfunctional and are likely re-wired during the acquisition of totipotency (Fig. 5B). Supporting this idea, a recent study demonstrates that the LEC2 activates RNA-dependent DNA methylation pathways to alter the epigenomic state (Peng et al., 2025). In summary, both cases of stoma-in-stoma and LEC2-SPCH-triggered somatic embryogenesis (Fig. 5) highlight that dynamic changes of epigenetic modifications, which decouple cell cycle from cell fate, underlie the key theme in cellular reprogramming.

## Conclusions and future perspectives

Over the past few years, the plant development and plant cell cycle fields have progressed rapidly, aided by new tools and approaches. This body of work, which we review here, reveals how the cell cycle interfaces with development. Developmental regulators directly control cell cycle timing, and cell cycle regulators can directly influence cell fate.

Recent insights into the relationship between cell cycle and cell identity in both the shoot and root were enabled by deep characterization of these developmental systems at a high spatiotemporal resolution, i.e. using scRNA-seq and advanced microscopy (Lee et al., 2025; Shahan et al., 2022; Tang et al., 2025; Vukašinović et al., 2025; Winter et al., 2024). Continued characterization of these developmental pathways has great potential to provide further insights into cell cycle development interactions. For example, with the advancement of single cell technologies, we may be able to map the cell cycle-specific epigenomic dynamics, which can be coupled with functional perturbations using chemical biology and genome-wide gene perturbation (e.g. CRISPR and/or RNAi library screen) to uncover the key regulatory events coupling cell cycle machineries to cell fate specification.

Crucially, cell cycle regulation largely occurs at the protein level. Yet our ability to assay protein levels and post-translational modifications, such as phosphorylation and ubiquitylation, in single cells or specific cell types has lagged behind our ability to measure mRNA transcripts. Future efforts to improve proteomic technologies have the potential to transform our understanding of the relationship between cell cycle and developmental processes. A gold standard for assessing the proteomic basis of the relationship between developmental and cell cycle regulation requires the production of cell type- and cell cycle phase-specific proteomics datasets. This is currently technically challenging due to low sample availability, detection limits and the difficulty of isolating plant cells by phase or type. Strategies involving phase-specific proteomics in animal cell lines found that thousands of proteins vary in terms of abundance and phosphorylation over the course of the cell cycle (Rega et al., 2025) and such a dataset would be valuable for the plant community.

Another approach would be to sort cells into plates, recording the ploidy of every sorted cell, and then proceed with single cell proteomics to gain insight into cell cycle regulation on a per cell basis. Single cell proteomics approaches are actively being developed in plants (Clark et al., 2022), but this remains technically challenging. Recent work in human cells combined scRNA-seq with the alternative approach of imaging proteomics to find a large number of cell cycle-regulated proteins that were previously uncharacterized are also largely regulated by kinase activity (Mahdessian et al., 2021). As such, imaging proteomics might be a good way to approach this problem in certain plant organs where cells are large enough for the spatial resolution of imaging proteomics. It is also important to specifically measure changes in phosphorylation status over the cell cycle, and active work on developing phosphoproteomics in plants (Duan et al., 2024) may soon make such studies feasible.

Dynamic protein complexes also regulate key cell cycle transitions, so mapping those complexes could greatly inform cell cycle research. Techniques like TurboID can be used to identify protein interactions in a time-gated manner in plants (Mair et al., 2019). Recently, a split TurboID system was developed that only functions when a complex of interest is assembled (Huang et al., 2025). This may be an especially useful tool for cell cycle proteomics characterization.

Moreover, advancements in metabolomic technologies will also be important to further our understanding of the relationship between cell cycle and development, especially in the context of the rapid responses of plant cell cycles and developmental processes to environmental perturbations. The immediate consequences of environmental challenges are likely to occur at the protein and metabolite levels, as is the case with GSH localization following root tip injury (Lee et al., 2025). Therefore, the development of proteomic and metabolomic tools could establish new avenues of inquiry into the relationship between cell cycle and development.

Finally, a clear trend has emerged from current literature that a core feature of stem cells is their unique cell cycle properties. In metazoans, rapid divisions are a hallmark of induced pluripotent stem cells and embryonic stem cells (Kapinas et al., 2013). In roots, stem cell initials divide slowly, while fate-specified cells go through a period of rapid, amplifying divisions followed by cell cycle exit. However, in the stomatal lineage, the phase of proliferative multipotent divisions occurs more quickly than the final, unipotent symmetric division. This inspires a question: which cells are truly stem cells and can their cell cycle behaviour serve as an indicator of stemness in plants? It might be possible that the bipotent stomatal meristemoid represents a ‘transient amplifying’ cell rather than a ‘stem’ cell. Perhaps, using the LEC2-induced totipotency system coupled with time-lapse live imaging to characterize cell cycle dynamics might address the intricate coupling of cell cycle regulation to stemness in plant cells across different species and developmental contexts. Addressing these questions will continue an inspiring tradition of plant cell cycle research.

## References

[DEV205318C1] Adrian, J., Chang, J., Ballenger, C. E., Bargmann, B. O. R., Alassimone, J., Davies, K. A., Lau, O. S., Matos, J. L., Hachez, C., Lanctot, A. et al. (2015). Transcriptome dynamics of the stomatal lineage: birth, amplification, and termination of a self- renewing population. *Dev. Cell* 33, 107-118. 10.1016/j.devcel.2015.01.025 25850675 PMC4390738

[DEV205318C2] Aida, M., Beis, D., Heidstra, R., Willemsen, V., Blilou, I., Galinha, C., Nussaume, L., Noh, Y.-S., Amasino, R. and Scheres, B. (2004). The PLETHORA genes mediate patterning of the Arabidopsis root stem cell niche. *Cell* 119, 109-120. 10.1016/j.cell.2004.09.018 15454085

[DEV205318C3] Arai, Y., Pulvers, J. N., Haffner, C., Schilling, B., Nüsslein, I., Calegari, F. and Huttner, W. B. (2011). Neural stem and progenitor cells shorten S-phase on commitment to neuron production. *Nat. Commun.* 2, 154. 10.1038/ncomms1155 21224845 PMC3105305

[DEV205318C4] Barlow, P. (1974). Regeneration of the cap of primary roots of *ZEA MAYS*. *New Phytol.* 73, 937-954. 10.1111/j.1469-8137.1974.tb01323.x

[DEV205318C5] Berckmans, B., Kirschner, G., Gerlitz, N., Stadler, R. and Simon, R. (2020). CLE40 signaling regulates root stem cell fate. *Plant Physiol.* 182, 1776-1792. 10.1104/pp.19.00914 31806736 PMC7140941

[DEV205318C6] Betegón-Putze, I., Mercadal, J., Bosch, N., Planas-Riverola, A., Marquès-Bueno, M., Vilarrasa-Blasi, J., Frigola, D., Burkart, R. C., Martínez, C., Conesa, A. et al. (2021). Precise transcriptional control of cellular quiescence by BRAVO/WOX5 complex in Arabidopsis roots. *Mol. Syst. Biol.* 17, e9864. 10.15252/msb.20209864 34132490 PMC8207686

[DEV205318C7] Boniotti, M. B. and Gutierrez, C. (2001). A cell-cycle-regulated kinase activity phosphorylates plant retinoblastoma protein and contains, in Arabidopsis, a CDKA/cyclin D complex: phosphorylation of plant retinoblastoma protein. *Plant J.* 28, 341-350. 10.1046/j.1365-313X.2001.01160.x 11722776

[DEV205318C8] Boudolf, V., Barrôco, R., Engler, J. A., Verkest, A., Beeckman, T., Naudts, M., Inzé, D. and De Veylder, L. (2004). B1-type cyclin-dependent kinases are essential for the formation of stomatal complexes in Arabidopsis thaliana. *Plant Cell* 16, 945-955. 10.1105/tpc.021774 15031414 PMC412868

[DEV205318C9] Boudolf, V., Inze, D. and De Veylder, L. (2006). What if higher plants lack a CDC25 phosphatase? *Trends Plant Sci.* 11, 474-479. 10.1016/j.tplants.2006.08.009 16949857

[DEV205318C10] Boudolf, V., Lammens, T., Boruc, J., Van Leene, J., Van Den Daele, H., Maes, S., Van Isterdael, G., Russinova, E., Kondorosi, E., Witters, E. et al. (2009). CDKB1;1 forms a functional complex with CYCA2;3 to suppress endocycle onset. *Plant Physiol.* 150, 1482-1493. 10.1104/pp.109.140269 19458112 PMC2705057

[DEV205318C11] Boutros, R., Dozier, C. and Ducommun, B. (2006). The when and wheres of CDC25 phosphatases. *Curr. Opin. Cell Biol.* 18, 185-191. 10.1016/j.ceb.2006.02.003 16488126

[DEV205318C12] Burian, A., Barbier de Reuille, P. and Kuhlemeier, C. (2016). Patterns of stem cell divisions contribute to plant longevity. *Curr. Biol.* 26, 1385-1394. 10.1016/j.cub.2016.03.067 27161504

[DEV205318C13] Churchman, M. L., Brown, M. L., Kato, N., Kirik, V., Hülskamp, M., Inzé, D., De Veylder, L., Walker, J. D., Zheng, Z., Oppenheimer, D. G. et al. (2006). SIAMESE, a plant-specific cell cycle regulator, controls endoreplication onset in Arabidopsis thaliana. *Plant Cell* 18, 3145-3157. 10.1105/tpc.106.044834 17098811 PMC1693949

[DEV205318C14] Clark, N. M., Elmore, J. M. and Walley, J. W. (2022). To the proteome and beyond: advances in single-cell omics profiling for plant systems. *Plant Physiol.* 188, 726-737. 10.1093/plphys/kiab429 35235661 PMC8825333

[DEV205318C15] Clowes, F. A. L. (1959). Reorganization of root apices after irradiation. *Ann. Bot.* 23, 205-210. 10.1093/oxfordjournals.aob.a083647

[DEV205318C16] Coronado, D., Godet, M., Bourillot, P.-Y., Tapponnier, Y., Bernat, A., Petit, M., Afanassieff, M., Markossian, S., Malashicheva, A., Iacone, R. et al. (2013). A short G1 phase is an intrinsic determinant of naïve embryonic stem cell pluripotency. *Stem Cell Res.* 10, 118-131. 10.1016/j.scr.2012.10.004 23178806

[DEV205318C17] Cruz-Ramírez, A., Díaz-Triviño, S., Blilou, I., Grieneisen, V. A., Sozzani, R., Zamioudis, C., Miskolczi, P., Nieuwland, J., Benjamins, R., Dhonukshe, P. et al. (2012). A bistable circuit involving SCARECROW-RETINOBLASTOMA integrates cues to inform asymmetric stem cell division. *Cell* 150, 1002-1015. 10.1016/j.cell.2012.07.017 22921914 PMC3500399

[DEV205318C18] Cruz-Ramírez, A., Díaz-Triviño, S., Wachsman, G., Du, Y., Arteága-Vázquez, M., Zhang, H., Benjamins, R., Blilou, I., Neef, A. B., Chandler, V. et al. (2013). A SCARECROW-RETINOBLASTOMA protein network controls protective quiescence in the Arabidopsis root stem cell organizer. *PLoS Biol.* 11, e1001724. 10.1371/journal.pbio.1001724 24302889 PMC3841101

[DEV205318C19] Cui, H. (2016). Middle cortex formation in the root: an emerging picture of integrated regulatory mechanisms. *Mol. Plant* 9, 771-773. 10.1016/j.molp.2016.05.002 27212386

[DEV205318C20] Dahl, M., Meskiene, I., Bögre, L., Ha, D. T., Swoboda, I., Hubmann, R., Hirt, H. and Heberle-Bors, E. (1995). The D-type alfalfa cyclin gene cycMs4 complements G1 cyclin-deficient yeast and is induced in the G1 phase of the cell cycle. *Plant Cell* 7, 1847-1857. 10.1105/tpc.7.11.1847 8535138 PMC161043

[DEV205318C21] Dalton, S. (2015). Linking the cell cycle to cell fate decisions. *Trends Cell Biol.* 25, 592-600. 10.1016/j.tcb.2015.07.007 26410405 PMC4584407

[DEV205318C22] D'Ario, M., Tavares, R., Schiessl, K., Desvoyes, B., Gutierrez, C., Howard, M. and Sablowski, R. (2021). Cell size controlled in plants using DNA content as an internal scale. *Science* 372, 1176-1181. 10.1126/science.abb4348 34112688

[DEV205318C23] De Veylder, L. (2019). The discovery of plant D-type cyclins. *Plant Cell* 31, 1194-1195. 10.1105/tpc.19.00277 31036595 PMC6588313

[DEV205318C24] De Veylder, L., Beeckman, T., Beemster, G. T., Krols, L., Terras, F., Landrieu, I., van der Schueren, E., Maes, S., Naudts, M. and Inzé, D. (2001). Functional analysis of cyclin-dependent kinase inhibitors of Arabidopsis. *Plant Cell* 13, 1653-1668. 10.1105/TPC.010087 11449057 PMC139548

[DEV205318C25] De Veylder, L., Beeckman, T. and Inzé, D. (2007). The ins and outs of the plant cell cycle. *Nat. Rev. Mol. Cell Biol.* 8, 655-665. 10.1038/nrm2227 17643126

[DEV205318C26] Desvoyes, B., Arana-Echarri, A., Barea, M. D. and Gutierrez, C. (2020). A comprehensive fluorescent sensor for spatiotemporal cell cycle analysis in Arabidopsis. *Nature Plants* 6, 1330-1334. 10.1038/s41477-020-00770-4 32989288

[DEV205318C27] Dewitte, W., Scofield, S., Alcasabas, A. A., Maughan, S. C., Menges, M., Braun, N., Collins, C., Nieuwland, J., Prinsen, E., Sundaresan, V. et al. (2007). Arabidopsis CYCD3 D-type cyclins link cell proliferation and endocycles and are rate-limiting for cytokinin responses. *Proc. Natl. Acad. Sci. USA* 104, 14537-14542. 10.1073/pnas.0704166104 17726100 PMC1964848

[DEV205318C28] Di Laurenzio, L., Wysocka-Diller, J., Malamy, J. E., Pysh, L., Helariutta, Y., Freshour, G., Hahn, M. G., Feldmann, K. A. and Benfey, P. N. (1996). The SCARECROW gene regulates an asymmetric cell division that is essential for generating the radial organization of the Arabidopsis root. *Cell* 86, 423-433. 10.1016/S0092-8674(00)80115-4 8756724

[DEV205318C29] Diaz Vivancos, P., Wolff, T., Markovic, J., Pallardó, F. V. and Foyer, C. H. (2010). A nuclear glutathione cycle within the cell cycle. *Biochem. J.* 431, 169-178. 10.1042/BJ20100409 20874710

[DEV205318C30] Dissmeyer, N., Weimer, A. K., De Veylder, L., Novak, B. and Schnittger, A. (2010). The regulatory network of cell-cycle progression is fundamentally different in plants versus yeast or metazoans. *Plant Signal. Behav.* 5, 1613-1618. 10.4161/psb.5.12.13969 21139435 PMC3115114

[DEV205318C31] Dolan, L., Janmaat, K., Willemsen, V., Linstead, P., Poethig, S., Roberts, K. and Scheres, B. (1993). Cellular organisation of the Arabidopsis thaliana root. *Development* 119, 71-84. 10.1242/dev.119.1.71 8275865

[DEV205318C32] Duan, X., Zhang, Y., Huang, X., Ma, X., Gao, H., Wang, Y., Xiao, Z., Huang, C., Wang, Z., Li, B. et al. (2024). GreenPhos, a universal method for in-depth measurement of plant phosphoproteomes with high quantitative reproducibility. *Mol. Plant* 17, 199-213. 10.1016/j.molp.2023.11.010 38018035

[DEV205318C33] Dubois, M., Achon, I., Brench, R. A., Polyn, S., Tenorio Berrío, R., Vercauteren, I., Gray, J. E., Inzé, D. and De Veylder, L. (2023). SIAMESE-RELATED1 imposes differentiation of stomatal lineage ground cells into pavement cells. *Nat. Plants* 9, 1143-1153. 10.1038/s41477-023-01452-7 37386150

[DEV205318C34] Echevarría, C., Desvoyes, B., Marconi, M., Franco-Zorrilla, J. M., Lee, L., Umeda, M., Sablowski, R., Birnbaum, K. D., Wabnik, K. and Gutierrez, C. (2025). Stem cell regulators drive a G1 duration gradient during plant root development. *Nat. Plants* 11, 2145-2155. 10.1038/s41477-025-02109-3 40968137 PMC12537498

[DEV205318C35] Edgar, B. A., Zielke, N. and Gutierrez, C. (2014). Endocycles: a recurrent evolutionary innovation for post-mitotic cell growth. *Nat. Rev. Mol. Cell Biol.* 15, 197-210. 10.1038/nrm3756 24556841

[DEV205318C36] Forzani, C., Aichinger, E., Sornay, E., Willemsen, V., Laux, T., Dewitte, W. and Murray, J. A. H. (2014). WOX5 suppresses CYCLIN D activity to establish quiescence at the center of the root stem cell niche. *Curr. Biol.* 24, 1939-1944. 10.1016/j.cub.2014.07.019 25127220 PMC4148176

[DEV205318C37] Fulcher, N. and Sablowski, R. (2009). Hypersensitivity to DNA damage in plant stem cell niches. *Proc. Natl. Acad. Sci. USA* 106, 20984-20988. 10.1073/pnas.0909218106 19933334 PMC2791609

[DEV205318C38] Fung, H. F., Amador, G. O., Dale, R., Gong, Y., Vollbrecht, M., Erberich, J. M., Mair, A. and Bergmann, D. C. (2025). Multi-scale dynamics influence the division potential of stomatal lineage ground cells in Arabidopsis. *Nat. Commun.* 16, 2612. 10.1038/s41467-025-57730-9 40097420 PMC11914061

[DEV205318C39] Glover, B. J. (2000). Differentiation in plant epidermal cells. *J. Exp. Bot.* 51, 497-505. 10.1093/jexbot/51.344.497 10938806

[DEV205318C40] Gombos, M., Raynaud, C., Nomoto, Y., Molnár, E., Brik-Chaouche, R., Takatsuka, H., Zaki, A., Bernula, D., Latrasse, D., Mineta, K. et al. (2023). The canonical E2Fs together with RETINOBLASTOMA-RELATED are required to establish quiescence during plant development. *Commun. Biol.* 6, 903. 10.1038/s42003-023-05259-2 37666980 PMC10477330

[DEV205318C41] Gong, Y., Dale, R., Fung, H. F., Amador, G. O., Smit, M. E. and Bergmann, D. C. (2023). A cell size threshold triggers commitment to stomatal fate in Arabidopsis. *Sci. Adv.* 9, eadf3497. 10.1126/sciadv.adf3497 37729402 PMC10881030

[DEV205318C42] Gutierrez, C. (2016). 25 years of cell cycle research: what's ahead? *Trends Plant Sci.* 21, 823-833. 10.1016/j.tplants.2016.06.007 27401252

[DEV205318C43] Gutierrez, C. (2022). A journey to the core of the plant cell cycle. *Int. J. Mol. Sci.* 23, 8154. 10.3390/ijms23158154 35897730 PMC9330084

[DEV205318C150] Gutzat, R., Borghi, L., Fütterer, J., Bischof, S., Laizet, Y., Hennig, L., Feil, R., Lunn, J. and Gruissem, W. (2011). RETINOBLASTOMA-RELATED PROTEIN controls the transition to autotrophic plant development. *Development* 138, 2977-2986. 10.1242/dev.060830 21693514

[DEV205318C44] Hachez, C., Ohashi-Ito, K., Dong, J. and Bergmann, D. C. (2011). Differentiation of Arabidopsis guard cells: analysis of the networks incorporating the basic helix-loop-helix transcription factor, FAMA. *Plant Physiol.* 155, 1458-1472. 10.1104/pp.110.167718 21245191 PMC3046599

[DEV205318C45] Han, S.-K. and Torii, K. U. (2019). Linking cell cycle to stomatal differentiation. *Curr. Opin. Plant Biol.* 51, 66-73. 10.1016/j.pbi.2019.03.010 31075538

[DEV205318C46] Han, S.-K., Qi, X., Sugihara, K., Dang, J. H., Endo, T. A., Miller, K. L., Kim, E.-D., Miura, T. and Torii, K. U. (2018). MUTE directly orchestrates cell-state switch and the single symmetric division to create stomata. *Dev. Cell* 45, 303-315.e5. 10.1016/j.devcel.2018.04.010 29738710

[DEV205318C47] Han, S.-K., Herrmann, A., Yang, J., Iwasaki, R., Sakamoto, T., Desvoyes, B., Kimura, S., Gutierrez, C., Kim, E.-D. and Torii, K. U. (2022). Deceleration of the cell cycle underpins a switch from proliferative to terminal divisions in plant stomatal lineage. *Dev. Cell* 57, 569-582.e6. 10.1016/j.devcel.2022.01.014 35148836 PMC8926846

[DEV205318C48] Helariutta, Y., Fukaki, H., Wysocka-Diller, J., Nakajima, K., Jung, J., Sena, G., Hauser, M. T. and Benfey, P. N. (2000). The SHORT-ROOT gene controls radial patterning of the Arabidopsis root through radial signaling. *Cell* 101, 555-567. 10.1016/S0092-8674(00)80865-X 10850497

[DEV205318C49] Heyman, J., Cools, T., Vandenbussche, F., Heyndrickx, K. S., Van Leene, J., Vercauteren, I., Vanderauwera, S., Vandepoele, K., De Jaeger, G., Van Der Straeten, D. et al. (2013). ERF115 controls root quiescent center cell division and stem cell replenishment. *Science* 342, 860-863. 10.1126/science.1240667 24158907

[DEV205318C50] Ho, C.-M. K., Bringmann, M., Oshima, Y., Mitsuda, N. and Bergmann, D. C. (2021). Transcriptional profiling reveals signatures of latent developmental potential in Arabidopsis stomatal lineage ground cells. *Proc. Natl. Acad. Sci. USA* 118, e2021682118. 10.1073/pnas.2021682118 33875598 PMC8092560

[DEV205318C51] Huang, A., Zhang, J., Liu, Z., Schoen, V., Verma, D., Zheng, H., Pedmale, U. V., Dong, J., de Lucas, M. and De Veylder, L. et al. (2025). Split-YFP-coupled interaction-dependent TurboID identifies new functions of basal cell polarity in Arabidopsis. *Proc. Natl. Acad. Sci. USA* 122, e2502445122. 10.1073/pnas.2502445122 40768356 PMC12358837

[DEV205318C52] Ikeuchi, M., Iwase, A., Rymen, B., Harashima, H., Shibata, M., Ohnuma, M., Breuer, C., Morao, A. K., de Lucas, M., De Veylder, L. et al. (2015). PRC2 represses dedifferentiation of mature somatic cells in Arabidopsis. *Nat. Plants* 1, 15089. 10.1038/nplants.2015.89 27250255

[DEV205318C53] Imai, K. K., Ohashi, Y., Tsuge, T., Yoshizumi, T., Matsui, M., Oka, A. and Aoyama, T. (2006). The A-type cyclin CYCA2;3 is a key regulator of ploidy levels in Arabidopsis endoreduplication. *Plant Cell* 18, 382-396. 10.1105/tpc.105.037309 16415207 PMC1356546

[DEV205318C54] Inzé, D. and De Veylder, L. (2006). Cell cycle regulation in plant development. *Annu. Rev. Genet.* 40, 77-105. 10.1146/annurev.genet.40.110405.090431 17094738

[DEV205318C55] Iwata, E., Ikeda, S., Matsunaga, S., Kurata, M., Yoshioka, Y., Criqui, M.-C., Genschik, P. and Ito, M. (2011). GIGAS CELL1, a novel negative regulator of the anaphase-promoting complex/cyclosome, is required for proper mitotic progression and cell fate determination in Arabidopsis. *Plant Cell* 23, 4382-4393. 10.1105/tpc.111.092049 22167058 PMC3269872

[DEV205318C56] Jang, J., Han, D., Golkaram, M., Audouard, M., Liu, G., Bridges, D., Hellander, S., Chialastri, A., Dey, S. S., Petzold, L. R. et al. (2019). Control over single-cell distribution of G1 lengths by WNT governs pluripotency. *PLoS Biol.* 17, e3000453. 10.1371/journal.pbio.3000453 31557150 PMC6782112

[DEV205318C57] Jiang, D. and Berger, F. (2017). DNA replication–coupled histone modification maintains Polycomb gene silencing in plants. *Science* 357, 1146-1149. 10.1126/science.aan4965 28818970

[DEV205318C58] John, P. C., Sek, F. J. and Lee, M. G. (1989). A homolog of the cell cycle control protein p34cdc2 participates in the division cycle of Chlamydomonas, and a similar protein is detectable in higher plants and remote taxa. *Plant Cell* 1, 1185-1193. 10.1105/tpc.1.12.1185 2535538 PMC159854

[DEV205318C59] Johnston, A. J., Kirioukhova, O., Barrell, P. J., Rutten, T., Moore, J. M., Baskar, R., Grossniklaus, U. and Gruissem, W. (2010). Dosage-sensitive function of RETINOBLASTOMA RELATED and convergent epigenetic control are required during the Arabidopsis life cycle. *PLoS Genet.* 6, e1000988. 10.1371/journal.pgen.1000988 20585548 PMC2887464

[DEV205318C60] Kapinas, K., Grandy, R., Ghule, P., Medina, R., Becker, K., Pardee, A., Zaidi, S. K., Lian, J., Stein, J., van Wijnen, A. et al. (2013). The abbreviated pluripotent cell cycle. *J. Cell. Physiol.* 228, 9-20. 10.1002/jcp.24104 22552993 PMC3667593

[DEV205318C61] Kim, S., Park, J. S., Lee, J., Lee, K. K., Park, O. S., Choi, H. S., Seo, P. J., Cho, H. T., Frost, J. M., Fischer, R. L. et al. (2021). The DME demethylase regulates sporophyte gene expression, cell proliferation, differentiation, and meristem resurrection. *Proc. Natl. Acad. Sci. USA* 118, e2026806118. 10.1073/pnas.2026806118 34266952 PMC8307533

[DEV205318C62] Kim, E.-D., Dorrity, M. W., Fitzgerald, B. A., Seo, H., Sepuru, K. M., Queitsch, C., Mitsuda, N., Han, S.-K. and Torii, K. U. (2022). Dynamic chromatin accessibility deploys heterotypic cis/trans-acting factors driving stomatal cell-fate commitment. *Nat. Plants* 8, 1453-1466. 10.1038/s41477-022-01304-w 36522450 PMC9788986

[DEV205318C63] Kono, A., Umeda-Hara, C., Adachi, S., Nagata, N., Konomi, M., Nakagawa, T., Uchimiya, H. and Umeda, M. (2007). The Arabidopsis D-type cyclin CYCD4 controls cell division in the stomatal lineage of the hypocotyl epidermis. *Plant Cell* 19, 1265-1277. 10.1105/tpc.106.046763 17449809 PMC1913761

[DEV205318C64] Kumar, N. and Larkin, J. C. (2017). Why do plants need so many cyclin-dependent kinase inhibitors? *Plant Signal. Behav.* 12, e1282021. 10.1080/15592324.2017.1282021 28165885 PMC5351735

[DEV205318C65] Lai, L. B., Nadeau, J. A., Lucas, J., Lee, E.-K., Nakagawa, T., Zhao, L., Geisler, M. and Sack, F. D. (2005). The Arabidopsis R2R3 MYB proteins FOUR LIPS and MYB88 restrict divisions late in the stomatal cell lineage. *Plant Cell* 17, 2754-2767. 10.1105/tpc.105.034116 16155180 PMC1242270

[DEV205318C66] Lau, O. S., Davies, K. A., Chang, J., Adrian, J., Rowe, M. H., Ballenger, C. E. and Bergmann, D. C. (2014). Direct roles of SPEECHLESS in the specification of stomatal self-renewing cells. *Science* 345, 1605-1609. 10.1126/science.1256888 25190717 PMC4390554

[DEV205318C67] Lee, L. R. and Bergmann, D. C. (2019). The plant stomatal lineage at a glance. *J. Cell Sci.* 132, jcs228551. 10.1242/jcs.228551 31028153 PMC6503951

[DEV205318C68] Lee, L. R., Wengier, D. L. and Bergmann, D. C. (2019). Cell-type-specific transcriptome and histone modification dynamics during cellular reprogramming in the Arabidopsis stomatal lineage. *Proc. Natl. Acad. Sci. USA* 116, 21914-21924. 10.1073/pnas.1911400116 31594845 PMC6815143

[DEV205318C69] Lee, L. R., Guillotin, B., Rahni, R., Hutchison, C., Desvoyes, B., Gutierrez, C. and Birnbaum, K. D. (2025). Glutathione accelerates the cell cycle and cellular reprogramming in plant regeneration. *Dev. Cell* 60, 1153-1167.e6. 10.1016/j.devcel.2024.12.019 39755116 PMC12278113

[DEV205318C70] Liu, Y., Xu, M., Liang, N., Zheng, Y., Yu, Q. and Wu, S. (2017). Symplastic communication spatially directs local auxin biosynthesis to maintain root stem cell niche in Arabidopsis. *Proc. Natl. Acad. Sci. USA* 114, 4005-4010. 10.1073/pnas.1616387114 28348232 PMC5393224

[DEV205318C71] Liu, A., Mair, A., Matos, J. L., Vollbrecht, M., Xu, S. L. and Bergmann, D. C. (2024). bHLH transcription factors cooperate with chromatin remodelers to regulate cell fate decisions during Arabidopsis stomatal development. *PLoS Biol.* 22, e3002770. 10.1371/journal.pbio.3002770 39150946 PMC11357106

[DEV205318C72] Livanos, P. and Müller, S. (2019). Division plane establishment and cytokinesis. *Annu. Rev. Plant Biol.* 70, 239-267. 10.1146/annurev-arplant-050718-100444 30795703

[DEV205318C73] Lodha, M., Marco, C. F. and Timmermans, M. C. P. (2013). The ASYMMETRIC LEAVES complex maintains repression of KNOX homeobox genes via direct recruitment of Polycomb-repressive complex2. *Genes Dev.* 27, 596-601. 10.1101/gad.211425.112 23468429 PMC3613607

[DEV205318C74] Lopez-Anido, C. B., Vatén, A., Smoot, N. K., Sharma, N., Guo, V., Gong, Y., Anleu Gil, M. X., Weimer, A. K. and Bergmann, D. C. (2021). Single-cell resolution of lineage trajectories in the Arabidopsis stomatal lineage and developing leaf. *Dev. Cell* 56, 1043-1055.e4. 10.1016/j.devcel.2021.03.014 33823130 PMC8054824

[DEV205318C75] Lui, H., Wang, H., Delong, C., Fowke, L. C., Crosby, W. L. and Fobert, P. R. (2000). The Arabidopsis Cdc2a-interacting protein ICK2 is structurally related to ICK1 and is a potent inhibitor of cyclin-dependent kinase activity in vitro. *Plant J.* 21, 379-385. 10.1046/j.1365-313x.2000.00688.x 10758489

[DEV205318C76] MacAlister, C. A., Ohashi-Ito, K. and Bergmann, D. C. (2007). Transcription factor control of asymmetric cell divisions that establish the stomatal lineage. *Nature* 445, 537-540. 10.1038/nature05491 17183265

[DEV205318C77] Mahdessian, D., Cesnik, A. J., Gnann, C., Danielsson, F., Stenström, L., Arif, M., Zhang, C., Le, T., Johansson, F., Schutten, R. et al. (2021). Spatiotemporal dissection of the cell cycle with single-cell proteogenomics. *Nature* 590, 649-654. 10.1038/s41586-021-03232-9 33627808

[DEV205318C78] Mair, A., Xu, S.-L., Branon, T. C., Ting, A. Y. and Bergmann, D. C. (2019). Proximity labeling of protein complexes and cell-type-specific organellar proteomes in Arabidopsis enabled by TurboID. *eLife* 8, e47864. 10.7554/eLife.47864 31535972 PMC6791687

[DEV205318C79] Marhava, P., Hoermayer, L., Yoshida, S., Marhavý, P., Benková, E. and Friml, J. (2019). Re-activation of stem cell pathways for pattern restoration in plant wound healing. *Cell* 177, 957-969.e13. 10.1016/j.cell.2019.04.015 31051107 PMC6506278

[DEV205318C80] Matos, J. L., Lau, O. S., Hachez, C., Cruz-Ramírez, A., Scheres, B. and Bergmann, D. C. (2014). Irreversible fate commitment in the Arabidopsis stomatal lineage requires a FAMA and RETINOBLASTOMA-RELATED module. *eLife* 3, e03271. 10.7554/eLife.03271 25303364 PMC4225492

[DEV205318C81] Matosevich, R. and Efroni, I. (2021). The quiescent center and root regeneration. *J. Exp. Bot.* 72, 6739-6745. 10.1093/jxb/erab319 34324634 PMC8513162

[DEV205318C82] Melaragno, J. E., Mehrotra, B. and Coleman, A. W. (1993). Relationship between endopolyploidy and cell size in epidermal tissue of Arabidopsis. *Plant Cell* 5, 1661-1668. 10.2307/3869747 12271050 PMC160394

[DEV205318C83] Menges, M. and Murray, J. A. H. (2002). Synchronous Arabidopsis suspension cultures for analysis of cell-cycle gene activity. *Plant J.* 30, 203-212. 10.1046/j.1365-313X.2002.01274.x 12000456

[DEV205318C84] Meyer, H. M., Teles, J., Formosa-Jordan, P., Refahi, Y., San-Bento, R., Ingram, G., Jönsson, H., Locke, J. C. W. and Roeder, A. H. K. (2017). Fluctuations of the transcription factor atml1 generate the pattern of giant cells in the arabidopsis sepal. *eLife* 6, e19131. 10.7554/eLife.19131 28145865 PMC5333958

[DEV205318C85] Motte, H., Vanneste, S. and Beeckman, T. (2019). Molecular and environmental regulation of root development. *Annu. Rev. Plant Biol.* 70, 465-488. 10.1146/annurev-arplant-050718-100423 30822115

[DEV205318C86] Mozgova, I., Köhler, C. and Hennig, L. (2015). Keeping the gate closed: functions of the polycomb repressive complex PRC2 in development. *Plant J.* 83, 121-132. 10.1111/tpj.12828 25762111

[DEV205318C87] Nakatani, T., Lin, J., Ji, F., Ettinger, A., Pontabry, J., Tokoro, M., Altamirano-Pacheco, L., Fiorentino, J., Mahammadov, E., Hatano, Y. et al. (2022). DNA replication fork speed underlies cell fate changes and promotes reprogramming. *Nat. Genet.* 54, 318-327. 10.1038/s41588-022-01023-0 35256805 PMC8920892

[DEV205318C88] Nowack, M. K., Harashima, H., Dissmeyer, N., Zhao, X., Bouyer, D., Weimer, A. K., De Winter, F., Yang, F. and Schnittger, A. (2012). Genetic framework of cyclin-dependent kinase function in Arabidopsis. *Dev. Cell* 22, 1030-1040. 10.1016/j.devcel.2012.02.015 22595674

[DEV205318C89] Oakenfull, E. A., Riou-Khamlichi, C. and Murray, J. A. H. (2002). Plant D-type cyclins and the control of G1 progression. *Philos. Trans. R. Soc. Lond. B Biol. Sci.* 357, 749-760. 10.1098/rstb.2002.1085 12079670 PMC1692988

[DEV205318C90] Ohashi-Ito, K., Bergmann, D. C. and Bergmann, D. C. (2006). Arabidopsis FAMA controls the final proliferation/differentiation switch during stomatal development. *Plant Cell* 18, 2493-2505. 10.1105/tpc.106.046136 17088607 PMC1626605

[DEV205318C91] Otero, S., Desvoyes, B., Peiró, R. and Gutierrez, C. (2016). Histone H3 dynamics reveal domains with distinct proliferation potential in the Arabidopsis root. *Plant Cell* 28, 1361-1371. 10.1105/tpc.15.01003 27207857 PMC4944401

[DEV205318C92] Padgett, J. and Santos, S. D. M. (2020). From clocks to dominoes: lessons on cell cycle remodelling from embryonic stem cells. *FEBS Lett.* 594, 2031-2045. 10.1002/1873-3468.13862 32535913

[DEV205318C93] Peng, J., Zhang, Q., Tang, L. P., Xu, B. J., Laux, T., Zhang, X. S. and Su, Y. H. (2025). LEC2 induces somatic cell reprogramming through epigenetic activation of plant cell totipotency regulators. *Nat. Commun.* 16, 4185. 10.1038/s41467-025-59335-8 40328763 PMC12056068

[DEV205318C94] Peres, A., Churchman, M. L., Hariharan, S., Himanen, K., Verkest, A., Vandepoele, K., Magyar, Z., Hatzfeld, Y., Van Der Schueren, E., Beemster, G. T. S. et al. (2007). Novel plant-specific cyclin-dependent kinase inhibitors induced by biotic and abiotic stresses. *J. Biol. Chem.* 282, 25588-25596. 10.1074/jbc.M703326200 17599908

[DEV205318C95] Péret, B., De Rybel, B., Casimiro, I., Benková, E., Swarup, R., Laplaze, L., Beeckman, T. and Bennett, M. J. (2009). Arabidopsis lateral root development: an emerging story. *Trends Plant Sci.* 14, 399-408. 10.1016/j.tplants.2009.05.002 19559642

[DEV205318C96] Petricka, J. J., Winter, C. M. and Benfey, P. N. (2012). Control of Arabidopsis root development. *Annu. Rev. Plant Biol.* 63, 563-590. 10.1146/annurev-arplant-042811-105501 22404466 PMC3646660

[DEV205318C97] Pi, L., Aichinger, E., van der Graaff, E., Llavata-Peris, C. I., Weijers, D., Hennig, L., Groot, E. and Laux, T. (2015). Organizer-derived WOX5 signal maintains root columella stem cells through chromatin-mediated repression of CDF4 expression. *Dev. Cell* 33, 576-588. 10.1016/j.devcel.2015.04.024 26028217

[DEV205318C98] Pillitteri, L. J., Sloan, D. B., Bogenschutz, N. L. and Torii, K. U. (2007). Termination of asymmetric cell division and differentiation of stomata. *Nature* 445, 501-505. 10.1038/nature05467 17183267

[DEV205318C99] Pollard, T. D. and O'Shaughnessy, B. (2019). Molecular mechanism of cytokinesis. *Annu. Rev. Biochem.* 88, 661-689. 10.1146/annurev-biochem-062917-012530 30649923 PMC6588489

[DEV205318C100] Rahni, R. and Birnbaum, K. D. (2019). Week-long imaging of cell divisions in the Arabidopsis root meristem. *Plant Methods* 15, 30. 10.1186/s13007-019-0417-9 30988691 PMC6446972

[DEV205318C101] Rahni, R., Efroni, I. and Birnbaum, K. D. (2016). A case for distributed control of local stem cell behavior in plants. *Dev. Cell* 38, 635-642. 10.1016/j.devcel.2016.08.015 27676436 PMC5076558

[DEV205318C102] Rahni, R., Lee, L. R., Vissers, G., Suresh, I., Gorodokin, B. M., Ip, P.-L., Guillotin, B. and Birnbaum, K. D. (2026). Histone deacetylases and cell cycle regulators orchestrate cell identity transitions during Arabidopsis root regeneration. *Mol. Plant.* 10.1016/j.molp.2026.03.013 PMC13180275 41889170

[DEV205318C103] Rega, C., Tsitsa, I., Roumeliotis, T. I., Krystkowiak, I., Portillo, M., Yu, L., Vorhauser, J., Pines, J., Mansfeld, J., Choudhary, J. et al. (2025). High resolution profiling of cell cycle-dependent protein and phosphorylation abundance changes in non-transformed cells. *Nat. Commun.* 16, 2579. 10.1038/s41467-025-57537-8 40089461 PMC11910661

[DEV205318C104] Rico-Resendiz, F., Pri-Tal, O., Raia, P., Moretti, A., Chen, H., Yu, J., Broger, L., Fuchs, C., Hothorn, L. A., Loubery, S. et al. (2026). Plant Kelch phosphatases are Ser/Thr phosphatases involved in cell cycle regulation. *bioRxiv* 2026.01.06.697939. 10.64898/2026.01.06.697939 PMC13214054 42166246

[DEV205318C105] Robinson, D. O. and Roeder, A. H. (2015). Themes and variations in cell type patterning in the plant epidermis. *Curr. Opin. Genet. Dev.* 32, 55-65. 10.1016/j.gde.2015.01.008 25727387

[DEV205318C106] Rossi, V. and Varotto, S. (2002). Insights into the G1/S transition in plants. *Planta* 215, 345-356. 10.1007/s00425-002-0780-y 12111215

[DEV205318C107] Sablowski, R. and Gutierrez, C. (2022). Cycling in a crowd: coordination of plant cell division, growth, and cell fate. *Plant Cell* 34, 193-208. 10.1093/plcell/koab222 34498091 PMC8774096

[DEV205318C108] Salomoni, P. and Calegari, F. (2010). Cell cycle control of mammalian neural stem cells: putting a speed limit on G1. *Trends Cell Biol.* 20, 233-243. 10.1016/j.tcb.2010.01.006 20153966

[DEV205318C109] Satake, A., Imai, R., Fujino, T., Tomimoto, S., Ohta, K., Na'iem, M., Indrioko, S., Widiyatno, W., Purnomo, S., Mollá-Morales, A. et al. (2023). Somatic mutation rates scale with time not growth rate in long-lived tropical trees. *eLife* 12, RP88456. 10.7554/eLife.88456 PMC11498935 39441734

[DEV205318C110] Seller, C. A. and Schroeder, J. I. (2023). Distinct guard cell-specific remodeling of chromatin accessibility during abscisic acid- and CO2-dependent stomatal regulation. *Proc. Natl. Acad. Sci. USA* 120, e2310670120. 10.1073/pnas.2310670120 38113262 PMC10756262

[DEV205318C111] Sena, G. and Birnbaum, K. D. (2010). Built to rebuild: in search of organizing principles in plant regeneration. *Curr. Opin Genet. Dev.* 20, 460-465. 10.1016/j.gde.2010.04.011 20537526 PMC2931316

[DEV205318C112] Sena, G., Wang, X., Liu, H.-Y., Hofhuis, H. and Birnbaum, K. D. (2009). Organ regeneration does not require a functional stem cell niche in plants. *Nature* 457, 1150-1153. 10.1038/nature07597 19182776 PMC2649681

[DEV205318C113] Shahan, R., Hsu, C.-W., Nolan, T. M., Cole, B. J., Taylor, I. W., Greenstreet, L., Zhang, S., Afanassiev, A., Vlot, A. H. C., Schiebinger, G. et al. (2022). A single-cell Arabidopsis root atlas reveals developmental trajectories in wild-type and cell identity mutants. *Dev. Cell* 57, 543-560.e9. 10.1016/j.devcel.2022.01.008 35134336 PMC9014886

[DEV205318C114] Simmons, A. R., Davies, K. A., Wang, W., Liu, Z. and Bergmann, D. C. (2019). SOL1 and SOL2 regulate fate transition and cell divisions in the Arabidopsis stomatal lineage. *Development* 146, dev171066. 10.1242/dev.171066 30665887 PMC6382008

[DEV205318C115] Simonini, S. (2025). Regulation of cell cycle in plant gametes: when is the right time to divide? *Development* 152, dev204217. 10.1242/dev.204217 39831611 PMC11829769

[DEV205318C116] Soni, R., Carmichael, J. P., Shah, Z. H. and Murray, J. A. (1995). A family of cyclin D homologs from plants differentially controlled by growth regulators and containing the conserved retinoblastoma protein interaction motif. *Plant Cell* 7, 85-103. 10.1105/tpc.7.1.85 7696881 PMC160767

[DEV205318C117] Sozzani, R., Cui, H., Moreno-Risueno, M. A., Busch, W., Van Norman, J. M., Vernoux, T., Brady, S. M., Dewitte, W., Murray, J. A. H. and Benfey, P. N. (2010). Spatiotemporal regulation of cell-cycle genes by SHORTROOT links patterning and growth. *Nature* 466, 128-132. 10.1038/nature09143 20596025 PMC2967763

[DEV205318C118] Stone, S. L., Kwong, L. W., Yee, K. M., Pelletier, J., Lepiniec, L., Fischer, R. L., Goldberg, R. B. and Harada, J. J. (2001). LEAFY COTYLEDON2 encodes a B3 domain transcription factor that induces embryo development. *Proc. Natl. Acad. Sci. USA* 98, 11806-11811. 10.1073/pnas.201413498 11573014 PMC58812

[DEV205318C119] Stone, S. L., Braybrook, S. A., Paula, S. L., Kwong, L. W., Meuser, J., Pelletier, J., Hsieh, T.-F., Fischer, R. L., Goldberg, R. B. and Harada, J. J. (2008). Arabidopsis LEAFY COTYLEDON2 induces maturation traits and auxin activity: implications for somatic embryogenesis. *Proc. Natl. Acad. Sci. USA* 105, 3151-3156. 10.1073/pnas.0712364105 18287041 PMC2268600

[DEV205318C120] Strotmann, V. I. and Stahl, Y. (2021). At the root of quiescence: function and regulation of the quiescent center. *J. Exp. Bot.* 72, 6716-6726. 10.1093/jxb/erab275 34111273

[DEV205318C121] Strotmann, V. I., García-Gómez, M. L. and Stahl, Y. (2025). Root stem cell homeostasis in Arabidopsis involves cell-type specific transcription factor complexes. *EMBO Rep.* 26, 2323-2346. 10.1038/s44319-025-00422-8 40108407 PMC12069552

[DEV205318C122] Sun, Y., Dilkes, B. P., Zhang, C., Dante, R. A., Carneiro, N. P., Lowe, K. S., Jung, R., Gordon-Kamm, W. J. and Larkins, B. A. (1999). Characterization of maize (Zea mays L.) Wee1 and its activity in developing endosperm. *Proc. Natl. Acad. Sci. USA* 96, 4180-4185. 10.1073/pnas.96.7.4180 10097184 PMC22441

[DEV205318C123] Takahashi, I., Kojima, S., Sakaguchi, N., Umeda-Hara, C. and Umeda, M. (2010). Two Arabidopsis cyclin A3s possess G1 cyclin-like features. *Plant Cell Rep.* 29, 307-315. 10.1007/s00299-010-0817-9 20130883

[DEV205318C124] Tang, L. P., Zhai, L. M., Li, J., Gao, Y., Ma, Q. L., Li, R., Liu, Q. F., Zhang, W. J., Yao, W. J., Mu, B. et al. (2025). Time-resolved reprogramming of single somatic cells into totipotent states during plant regeneration. *Cell* 188, 7009-7015. 10.1016/j.cell.2025.10.035 41197625

[DEV205318C125] Timilsina, R., Kim, J. H., Nam, H. G. and Woo, H. R. (2019). Temporal changes in cell division rate and genotoxic stress tolerance in quiescent center cells of Arabidopsis primary root apical meristem. *Sci. Rep.* 9, 3599. 10.1038/s41598-019-40383-2 30837647 PMC6400898

[DEV205318C126] Torii, K. U. (2021). Stomatal development in the context of epidermal tissues. *Ann. Bot.* 128, 137-148. 10.1093/aob/mcab052 33877316 PMC8324025

[DEV205318C127] Trouth, A., Ravichandran, K., Gafken, P. R., Martire, S., Boyle, G. E., Veronezi, G. M. B., La, V., Namciu, S. J., Banaszynski, L. A., Sarthy, J. F. et al. (2025). The length of the G1 phase is an essential determinant of H3K27me3 landscapes across diverse cell types. *PLoS Biol.* 23, e3003119. 10.1371/journal.pbio.3003119 40245079 PMC12052206

[DEV205318C128] Tulin, F., Aizezi, Y., Reyes, A. V., Fujieda, Y., Grossman, A., Xu, S.-L., Onishi, M., Assaad, F. F. and Wang, Z.-Y. (2025). Mitotic entry is controlled by the plant-specific phosphatase BSL1 and cyclin-dependent kinase B. *Nat. Plants* 11, 2395-2408. 10.1038/s41477-025-02145-z 41233566 PMC12626892

[DEV205318C129] Uchida, N. and Torii, K. U. (2019). Stem cells within the shoot apical meristem: identity, arrangement and communication. *Cell. Mol. Life Sci.* 76, 1067-1080. 10.1007/s00018-018-2980-z 30523363 PMC11105333

[DEV205318C130] van den Berg, C., Willemsen, V., Hage, W., Weisbeek, P. and Scheres, B. (1995). Cell fate in the Arabidopsis root meristem determined by directional signalling. *Nature* 378, 62-65. 10.1038/378062a0 7477287

[DEV205318C131] van den Berg, C., Willemsen, V., Hendriks, G., Weisbeek, P. and Scheres, B. (1997). Short-range control of cell differentiation in the Arabidopsis root meristem. *Nature* 390, 287-289. 10.1038/36856 9384380

[DEV205318C132] Vandepoele, K., Raes, J., De Veylder, L., Rouzé, P., Rombauts, S. and Inzé, D. (2002). Genome-wide analysis of core cell cycle genes in Arabidopsis. *Plant Cell* 14, 903-916. 10.1105/tpc.010445 11971144 PMC150691

[DEV205318C133] Vanneste, S., Coppens, F., Lee, E., Donner, T. J., Xie, Z., Van Isterdael, G., Dhondt, S., De Winter, F., De Rybel, B., Vuylsteke, M. et al. (2011). Developmental regulation of CYCA2s contributes to tissue-specific proliferation in Arabidopsis: developmental control of CYCA2. *EMBO J.* 30, 3430-3441. 10.1038/emboj.2011.240 21772250 PMC3160660

[DEV205318C134] Vernoux, T., Wilson, R. C., Seeley, K. A., Reichheld, J. P., Muroy, S., Brown, S., Maughan, S. C., Cobbett, C. S., Van Montagu, M., Inzé, D. et al. (2000). The ROOT MERISTEMLESS1/CADMIUM SENSITIVE2 gene defines a glutathione-dependent pathway involved in initiation and maintenance of cell division during postembryonic root development. *Plant Cell* 12, 97-110. 10.1105/tpc.12.1.97 10634910 PMC140217

[DEV205318C135] Vilarrasa-Blasi, J., González-García, M.-P., Frigola, D., Fàbregas, N., Alexiou, K. G., López-Bigas, N., Rivas, S., Jauneau, A., Lohmann, J. U., Benfey, P. N. et al. (2014). Regulation of plant stem cell quiescence by a brassinosteroid signaling module. *Dev. Cell* 30, 36-47. 10.1016/j.devcel.2014.05.020 24981610

[DEV205318C136] Vukašinović, N., Hsu, C.-W., Marconi, M., Li, S., Zachary, C., Shahan, R., Szekley, P., Aardening, Z., Vanhoutte, I., Ma, Q. et al. (2025). Polarity-guided uneven mitotic divisions control brassinosteroid activity in proliferating plant root cells. *Cell* 188, 2063-2080.e24. 10.1016/j.cell.2025.02.011 40068682

[DEV205318C137] Wang, H., Fowke, L. C. and Crosby, W. L. (1997). A plant cyclin-dependent kinase inhibitor gene. *Nature* 386, 451-452. 10.1038/386451a0 9087400

[DEV205318C138] Wang, G., Kong, H., Sun, Y., Zhang, X., Zhang, W., Altman, N., DePamphilis, C. W. and Ma, H. (2004). Genome-wide analysis of the cyclin family in Arabidopsis and comparative phylogenetic analysis of plant cyclin-like proteins. *Plant Physiol.* 135, 1084-1099. 10.1104/pp.104.040436 15208425 PMC514142

[DEV205318C139] Weimer, A. K., Nowack, M. K., Bouyer, D., Zhao, X., Harashima, H., Naseer, S., De Winter, F., Dissmeyer, N., Geldner, N. and Schnittger, A. (2012). RETINOBLASTOMA RELATED1 regulates asymmetric cell divisions in Arabidopsis. *Plant Cell* 24, 4083-4095. 10.1105/tpc.112.104620 23104828 PMC3517237

[DEV205318C140] Weimer, A. K., Matos, J. L., Sharma, N., Patell, F., Murray, J. A. H., Dewitte, W. and Bergmann, D. C. (2018). Lineage- and stage-specific expressed CYCD7;1 coordinates the single symmetric division that creates stomatal guard cells. *Development* 145, dev160671. 10.1242/dev.160671 29467245 PMC5897600

[DEV205318C141] Willems, A. and De Veylder, L. (2022). The plant anaphase-promoting complex/cyclosome. *Annu. Rev. Cell Dev. Biol.* 38, 25-48. 10.1146/annurev-cellbio-120420-092421 35395166

[DEV205318C142] Winter, C. M., Szekely, P., Popov, V., Belcher, H., Carter, R., Jones, M., Fraser, S. E., Truong, T. V. and Benfey, P. N. (2024). SHR and SCR coordinate root patterning and growth early in the cell cycle. *Nature* 626, 611-616. 10.1038/s41586-023-06971-z 38297119 PMC10866714

[DEV205318C143] Xie, Z., Lee, E., Lucas, J. R., Morohashi, K., Li, D., Murray, J. A. H., Sack, F. D. and Grotewold, E. (2010). Regulation of cell proliferation in the stomatal lineage by the Arabidopsis MYB FOUR LIPS via direct targeting of core cell cycle genes. *Plant Cell* 22, 2306-2321. 10.1105/tpc.110.074609 20675570 PMC2929110

[DEV205318C144] Yamamuro, C., Miki, D., Zheng, Z., Ma, J., Wang, J., Yang, Z., Dong, J. and Zhu, J. K. (2014). Overproduction of stomatal lineage cells in Arabidopsis mutants defective in active DNA demethylation. *Nat. Commun.* 5, 4062. 10.1038/ncomms5062 24898766 PMC4097119

[DEV205318C145] Yang, K., Wang, H., Xue, S., Qu, X., Zou, J. and Le, J. (2014). Requirement for A-type cyclin-dependent kinase and cyclins for the terminal division in the stomatal lineage of Arabidopsis. *J. Exp. Bot.* 65, 2449-2461. 10.1093/jxb/eru139 24687979 PMC4036514

[DEV205318C146] Yang, K.-Z., Jiang, M., Wang, M., Xue, S., Zhu, L.-L., Wang, H.-Z., Zou, J.-J., Lee, E.-K., Sack, F. and Le, J. (2015). Phosphorylation of Serine 186 of bHLH transcription factor SPEECHLESS promotes stomatal development in Arabidopsis. *Mol. Plant* 8, 783-795. 10.1016/j.molp.2014.12.014 25680231

[DEV205318C147] Yang, H., Berry, S., Olsson, T. S. G., Hartley, M., Howard, M. and Dean, C. (2017). Distinct phases of Polycomb silencing to hold epigenetic memory of cold in Arabidopsis. *Science* 357, 1142-1145. 10.1126/science.aan1121 28818969

[DEV205318C148] Zuch, D. T., Doyle, S. M., Majda, M., Smith, R. S., Robert, S. and Torii, K. U. (2022). Cell biology of the leaf epidermis: fate specification, morphogenesis, and coordination. *Plant Cell* 34, 209-227. 10.1093/plcell/koab250 34623438 PMC8774078

[DEV205318C149] Zuch, D. T., Herrmann, A., Kim, E.-D. and Torii, K. U. (2023). Cell cycle dynamics during stomatal development: window of MUTE action and ramification of its loss-of-function on an uncommitted precursor. *Plant Cell Physiol.* 64, 325-335. 10.1093/pcp/pcad002 36609867 PMC10016323

